# Geometric phase sensing using seismic waves for comprehensive volcano monitoring at Kı̄lauea Hawaii

**DOI:** 10.1038/s41467-026-73998-x

**Published:** 2026-06-26

**Authors:** Bingxu Luo, Susan Beck, Pierre Deymier, Keith Runge, Eric Kiser, Pranabendu Moitra, Falk Huettmann, Samy Missoum, Marat Latypov, Elizabeth Whitney, Jiayang Wang, Sinan S. Schabib

**Affiliations:** 1 New Frontiers of Sound Science and Technology Center, The University of Arizona, Tucson, AZ USA; 2 Department of Geosciences, The University of Arizona, Tucson, AZ USA; 3 Department of Materials Science and Engineering, The University of Arizona, Tucson, AZ USA; 4 -EWHALE Lab-. Department of Biology and Wildlife. Institute of Arctic Biology, University of Alaska Fairbanks, Fairbanks, AK USA; 5 Department of Aerospace and Mechanical Engineering, The University of Arizona, Tucson, AZ USA

**Keywords:** Seismology, Volcanology

## Abstract

Monitoring volcanic activity requires sensitive tools capable of detecting subtle subsurface changes across eruptive cycles. While seismic methods are widely used to study volcanic unrest, few provide continuous tracking throughout eruptive stages with clearly interpretable signals. We present a geometric phase sensing approach, rooted in topological acoustics, that encodes the intrinsic geometry of the seismic wavefield. By integrating signals across a station array, the geometric phase change (Δ*η*) captures wavepath-integrated medium perturbations, providing a stable measure of evolving subsurface conditions distinct from conventional waveform coherence metrics. Applied to Kı̄lauea volcano, Δ*η* closely tracks precursory magmatic pressurization and co-eruptive caldera collapse during the 2018 eruption, identifies two major intrusion events and post-eruptive recovery spanning five eruptions from 2020 to 2024. Δ*η* exhibits systematic and interpretable signatures of eruptive transitions, even under strongly perturbed ambient noise conditions. Numerical simulations corroborate these observations, indicating a sensitivity of  ~15.0% per MPa to subsurface pressure changes. By leveraging geometric phase approach, we establish a monitoring framework applicable to volcanic and other dynamic Earth systems.

## Abstract (web summary)

This study introduces geometric phase sensing, a topological acoustics framework using seismic noise to track volcanic activity at Kīlauea. The method reveals subsurface changes over the full eruptive stages and enables monitoring across diverse dynamic Earth systems.

## Introduction

Volcanoes are major natural hazards that threaten over 500 million people worldwide, with risks growing as populations and infrastructure expand near active systems^1^. Mitigating these hazards requires monitoring the full evolution of volcanic activity from subtle magmatic unrest to co-eruptive fracturing and collapse, and eventual recovery. This task remains highly challenging yet essential for identifying eruption triggers and improving hazard assessment. Among geophysical volcano monitoring techniques, seismology stands out by providing continuous, high-resolution records of ground motion signals, offering a powerful window for probing subsurface dynamics. Over the past decades, advances in ambient seismic noise interferometry have enabled the empirical approximation of Green’s functions (i.e., the effective signal responses of the medium) by cross-correlating noise recordings from seismic stations^2–7^. This approach has facilitated passive seismic monitoring techniques, such as relative seismic velocity changes (*dv*/*v*)^8,9^, which measure dynamical phase shifts (i.e., time shifts) between pairwise cross-correlated waveforms. This seismic velocity sensing has proven effective in detecting subsurface medium variations and therefore has been widely applied to track volcanic dynamics^10–20^. However, this dynamical phase-based method relies on high coherence between waveforms, which always requires a stable ambient noise field to achieve reliable measurements^4,21,22^. This aspect faces substantial challenges when highly variable volcanic source signals degrade the waveform coherence over long-term volcanic evolutions. In addition, complex processes of volcanic edifices can induce diverse seismic velocity changes, complicating the interpretation of observations^14,15,19,20^. These limitations are particularly critical when both high sensitivity and reliable interpretability are required to anticipate hazardous volcanic activity, underscoring the need for approaches that better exploit information-rich signals embedded in ambient seismic noise.

Topological acoustics enables alternative ways of transporting information by leveraging topological features to control wave propagation^23,24^, going beyond conventional wave properties such as amplitude and dynamical phase. Central to topological acoustics is the concept of geometry^23^. In an acoustic field that contains the distribution of sound waves, geometry can be used to characterize the spatial field pattern. This is done by defining a coordinate system in the wave-supporting medium, and one can assign physical quantities (such as pressure amplitude) at each point to build a geometric representation of the sound field. Extending this geometry perspective to the ambient seismic field, the configuration of a subset of seismic stations defines a conceptual coordinate system in the medium. Approximated Green’s functions between station pairs provide quantities at points within this system, allowing us to sample a geometric representation of the cross-correlated seismic wavefield (hereafter referred to as the wavefield). Comparing two such wavefield representations obtained at different states gives rise to the concept of the geometric phase, which captures the evolution of the wavefield geometry and, in theory, the variations in the underlying medium. In practice, the wavefield geometry is represented by building a vector in space that incorporates both the array configuration and cross-correlations, and is projected as a state vector in an abstract Hilbert (parameter) space. Changes in the geometric phase (Δ*η*) can then be quantified by tracking the state vectors in the same Hilbert space, forming the basis of geometric phase sensing, a framework that has broad applications across the physical sciences for detecting subtle changes in complex wavefields and medium variations. The term “geometric phase" has previously been used in seismology to describe Berry phase-related phenomena^25^, including shear-wave polarization arising from cyclic evolution in periodic systems such as Earth’s self-rotation^26–28^. However, the geometric phase (Δ*η*) introduced here is conceptually different from the Berry-phase effect tied to cyclic or periodic evolution. It is rigorously defined as an operational, directly observable sensing quantity, representing an accumulated phase extracted from non-periodic ambient noise interferometric wavefields, therefore establishing a distinct physical and methodological framework.

Unlike the conventional seismic velocity sensing that measures dynamical phase shifts within each station pair, geometric phase sensing treats the entire wavefield by incorporating a subset of all stations, allowing full wavefield information to be captured in a single observable. This sensing method has been validated in numerical simulations^29,30^, where the geometric phase derived from simulated acoustic waves showed sensitivity to variations in global properties of the medium. Furthermore, geometric phase sensing has been successfully applied in non-destructive testing experiments in material science^31–33^, where ultrasonic waves with frequencies from tens to hundreds of kHz were used to detect structural mass defects at millimeter scales. These studies demonstrate the method’s high sensitivity to perturbations in the wave-supporting medium and highlight its application across multiple scientific disciplines.

Building on its success in acoustics, we advance geometric phase sensing with ambient seismic noise to address the challenge of tracking subtle subsurface changes in volcanic dynamics. We focus on Kı̄lauea volcano, Hawaii, one of the world’s most active basaltic volcanoes, whose well-documented eruptions and open-source seismic and geodetic networks provide a suitable natural laboratory. The eruptive processes observed at Kı̄lauea, including magma pressurization, caldera collapse, and post-eruptive recovery, are characteristic of many volcanic systems worldwide, allowing insights gained here to be evaluated in a setting relevant to other volcanic monitoring. In this study, we develop a geometric phase sensing framework that leverages ambient seismic noise to continuously track volcanic dynamics. By detecting subtle wavefield perturbations, this method reveals precursory subsurface pressurization, captures structural collapse, and documents post-eruptive recovery. Complementary numerical simulations further illuminate the sensitivity of the geometric phase to source variability and subsurface pressure perturbations. We evaluate geometric phase sensing as a framework for tracking volcanic processes, with potential applicability to environmental and geohazard systems.

## Results

### Constructing geometric phase sensing framework for the Kı̄lauea volcano

**Fig. 1:**
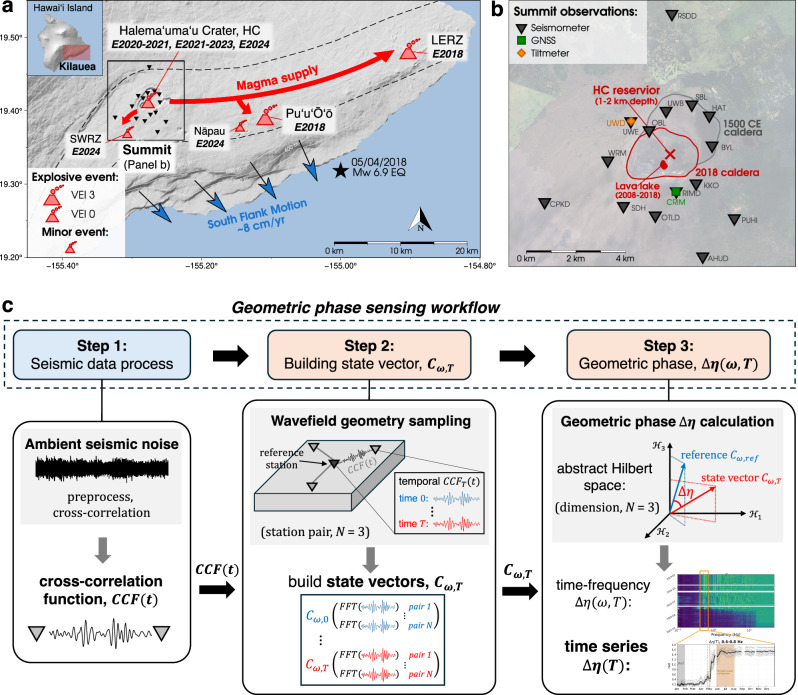
Study area and geometric phase sensing framework. **a** Mapview of Kı̄lauea volcano. Eruptions are labeled with their vent sites and years (e.g., LERZ ***E2018***). Explosive events are scaled by Volcanic Explosivity Index (VEI) values compiled from the Smithsonian Institution Global Volcanism Program (https://volcano.si.edu/). Maximum south flank motion (~8 cm/yr) is adapted from a published study^40^. LERZ Lower East Rift Zone, SWRZ Southwest Rift Zone. **b** Close-up view of the Kı̄lauea summit. Selected seismic stations (black triangles) and other geophysical observations are operated by the Hawaiian Volcano Observatory. The inferred depth of HC reservoir (1–2 km) is adapted from a published study^34^. **a**, **b** are plotted using the Generic Mapping Tool^68^. **c** Geometric phase sensing workflow. In Step 2, four example stations are shown, with the central one selected as the reference. In “Methods”, we detail the implementation of each step within the workflow.

Kı̄lauea volcano, located on the southeastern flank of Hawaii Island, has experienced multiple significant eruptions over the past decade. From 1983 to 2018, summit-supplied magma erupted nearly continuously from the Pu‘u‘Ō‘ō vents^34,35^ (Fig. 1a). The 2018 destructive eruption released around 1 km^3^ of erupted lava and incurred economic losses exceeding $800 million for long-term recovery^36–38^. During the 2018 eruption, ~825,000,000 m^3^ of summit edifice collapsed and formed a new caldera over the old one that was created in 1500 CE^34^ (Fig. 1b). Afterwards, at least five explosive and/or effusive eruptions were observed in the summit and rift zone through 2024 (Fig. 1a). The mantle-driven magma supply of Kı̄lauea largely passed through the summit reservoir system before migrating laterally down to multiple vents in the East and Southwest Rift Zone (Fig. 1a). There are two primary magma storage reservoirs at the summit: a shallower Halema‘uma‘u Crater (HC) reservoir centered at a depth of 1–2 km (Fig. 1b) and a deeper South Caldera reservoir^39^. Therefore, the summit area has been closely monitored over the years to assess magmatic processes and issue early warnings for potential volcanic hazards.

The Hawaiian Volcano Observatory (HVO) operates an open-source volcano monitoring network at Kı̄lauea summit (Fig. 1b), enabling us to implement geometric phase sensing and evaluate its performance against independent geophysical observations. Figure 1c illustrates the geometric phase sensing workflow. Step 1 preprocesses continuous seismic recordings and computes cross-correlation functions (CCFs) between station pairs. In Step 2, these cross-correlations are incorporated to build the state vectors sampling the evolving wavefield geometry. Step 3 extracts the geometric phase by tracking these state vectors in a multidimensional abstract Hilbert space, yielding its time- and frequency-dependent variations. From these, we derive geometric phase time series at specific frequencies or frequency bands. This workflow effectively transforms raw seismic recordings into geometric phase observables, allowing us to track volcanic dynamics through the full eruption chronology. Detailed implementation of each step is provided in “Methods”.

### Geometric phase tracking across multiple eruptions of Kı̄lauea from 2018 to 2024

**Fig. 2:**
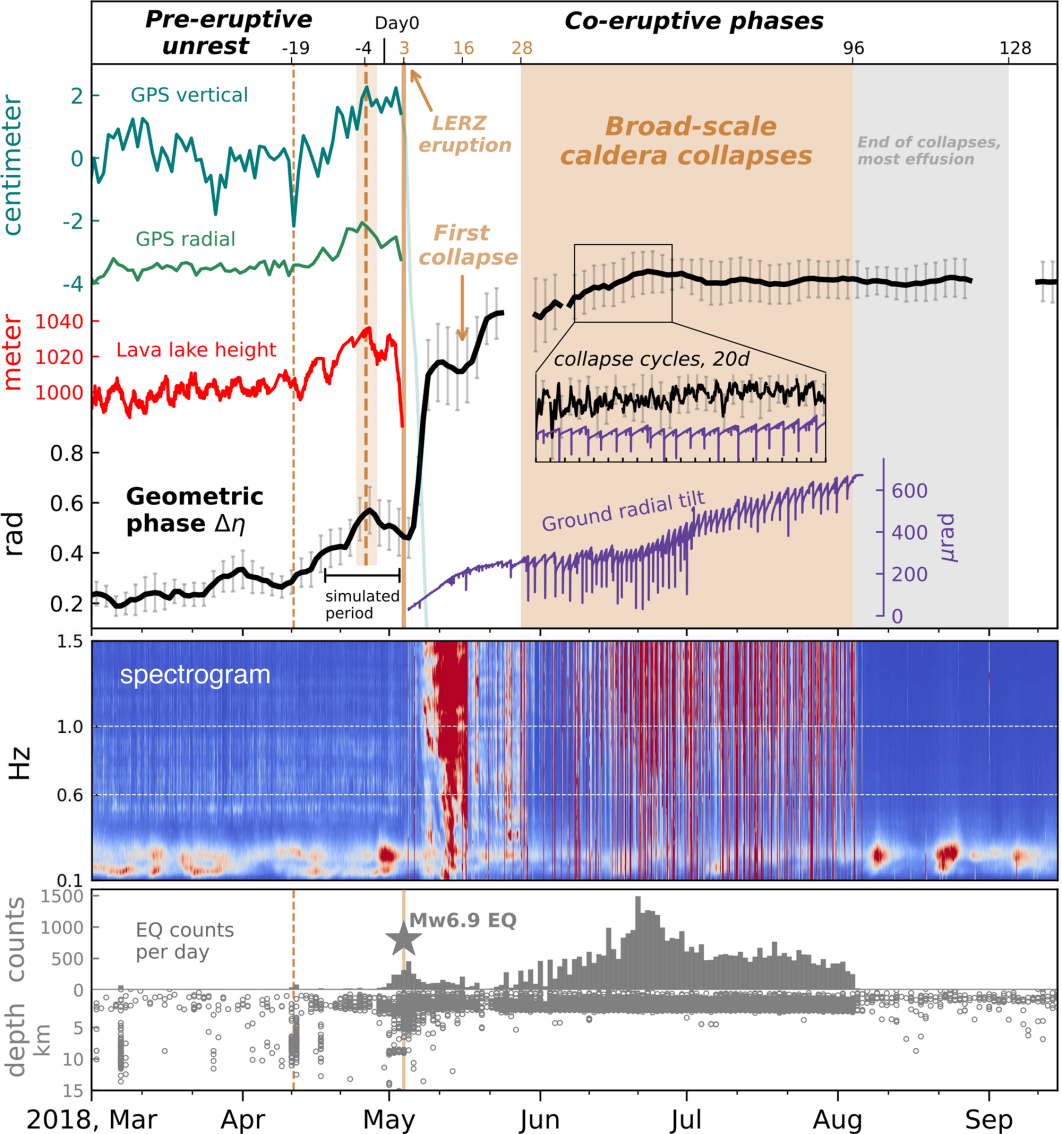
Time series of the geometric phase and observations during the 2018 Kı̄lauea eruption. Eruption days are calculated relative to the first fissure that opened at Pu‘u’Ō‘ō on April 30 (Day 0, Hawaiian Standard Time). The thick black curve represents the mean level of the geometric phase changes (Δ*η*) across all reference station choices, while the error bar denotes its standard deviation. The inset box displays a representative 20-day collapse window. GPS data is sourced from station CRIM of the Global Navigation Satellite System (GNSS). Ground tilt data is obtained from the tiltmeter UWD. The simulated time period (April 18 to May 3) is selected for the numerical simulation of Δ*η* (Fig. 5). The spectrogram is shown for the vertical components of station RIMD and has been smoothed using a Gaussian filter with two times the standard deviation. 0.6–1.0 Hz is denoted as the frequency range used for the Δ*η* analysis.

We examine geometric phase sensing by analyzing Kı̄lauea eruptions from 2018 to 2024. Figure 2 provides a detailed view of geometric phase (Δ*η*) time series, capturing the full sequence of the 2018 destructive eruption, and compared with independent observations. Day 0 corresponds to the first fissure that opened at Pu‘u’Ō‘ō on April 30, 2018 (Hawaiian Standard Time). Δ*η* begins to show several minor perturbations starting in March and experiences a significant increase from April 11 (Day −19), peaking on April 26 (Day −4) (Fig. 2). This pre-eruptive evolution of Δ*η* exhibits a consistent pattern of centimeters of surface ground inflation, tens of meters of lava lake rise, and several earthquake (EQ) swarms, indicating repeated deflation-inflation events due to accelerated pressurization of the magma reservoir^19,36,40^. The first transient increase in Δ*η* corresponds to the onset of the LERZ eruption on May 3 (Day 3) and the subsequent *M*_*W*_ (moment magnitude) 6.9 earthquake beneath Kı̄lauea’s south flank the following day (Fig. 2). At this time, the dramatic subsidence of the ground and withdrawal of the lava lake indicate depressurization following the rapid drainage of the magma reservoir from the summit^34^. The spectrogram panel reveals a pronounced broadband energy burst coincident with these co-eruptive transitions, with elevated power spanning the analyzed frequency band. The second transient increase in Δ*η* occurs in mid-May (Day 16), aligning with the onset of the summit caldera collapse after large-scale magma evacuation. The inset panel of Fig. 2 shows short-term (June 8–28) hourly perturbations of Δ*η* during the broad-scale caldera collapses, exhibiting an in-phase cycle with ground tilt and revealing subtle, nearly day-long collapse cycles. The full hourly evolution of Δ*η* is presented in Supplementary Fig. S1. On August 4 (Day 96), Δ*η* stabilizes at a constant level after its sustained evolution over four months, which corresponds with the completion of caldera collapse and most lava effusion. We emphasize that these co-eruptive Δ*η* signals are inherently non-unique and likely reflect coupled effects of temporally varying ambient noise fields and subsurface structural changes during eruptions. Further discussion is provided in the “Discussion” section.

**Fig. 3:**
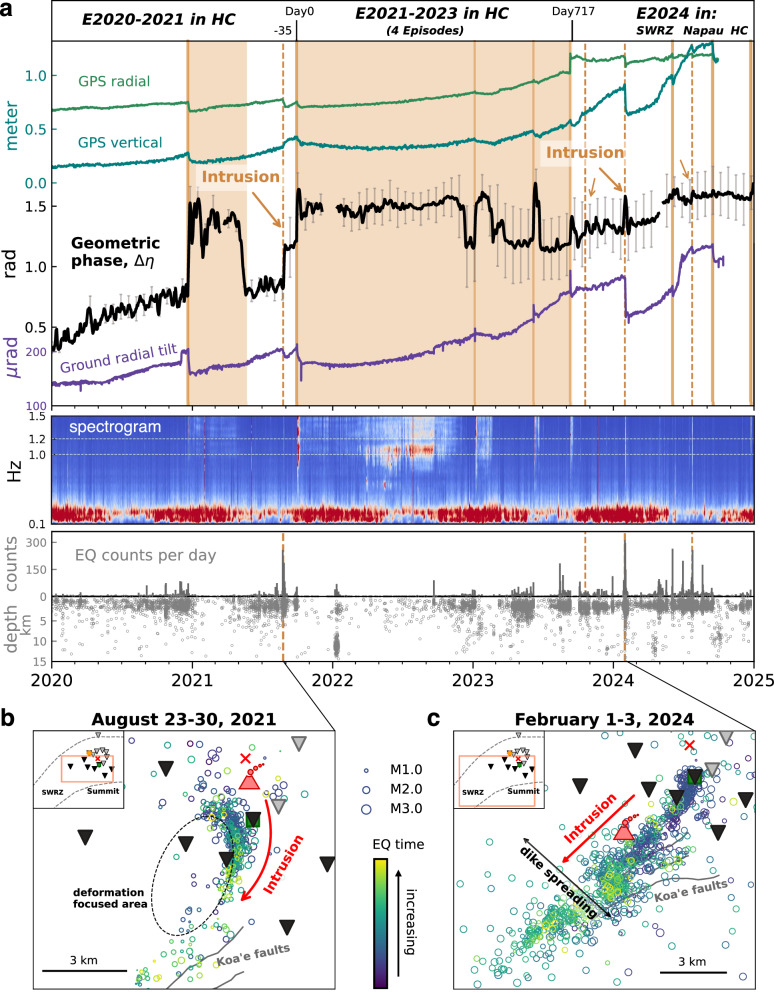
Time series of the geometric phase and observations during Kı̄lauea eruptions from 2020 to 2024. **a** Five eruptions are marked at the top; their onsets and durations are indicated by yellow solid lines and shaded bands. Four detected magma intrusions are shown with dashed lines. Other symbols are the same as in Fig. 2. **b**, **c** Map views of two major magma intrusions. Circles represent earthquakes, color-coded by their occurrence times and scaled by magnitude. Gray triangles mark seismic stations excluded from the 2020 to 2024 analysis due to key recording gaps. The red cross indicates the approximate location of HC reservoir. The two volcano symbols mark the eruption vents of E2021–2023 in HC and E2024 in SWRZ.

After a quasi-quiet year in 2019, five subsequent explosive and/or effusive eruptions occurred at Kı̄lauea from 2020 to 2024. A distinct feature is that, unlike remaining at a high amplitude level after the 2018 eruption, Δ*η* rapidly recovered to nearly pre-eruptive levels at the end of each eruption (Fig. 3a). In particular, this Δ*η* behavior successfully identified four eruptive episodes during E2021–2023 (Fig. 3a). Such rapid recovery of Δ*η* may serve as an eruption termination indicator, and we provide detailed investigations in the next section. Besides eruptive signatures, two significant magma intrusions, validated with ground deformation and confirmed in HVO reports^41,42^, are revealed as distinct perturbations in Δ*η* (highlighted by two bold arrows in Fig. 3a). During these two intrusions, the magma movements from the summit storage reservoir to the southwest are clearly delineated by the seismicity migration patterns (Fig. 3b, c). In February 2024, the magma extended farther to the SWRZ along the regional Koa’e faults (Fig. 3c), and a narrow band of subsidence marked where the dike (a vertical sheet of magma) intruded and then laterally spread^42^. Two additional minor magma intrusive periods in October 2023 and August 2024, reported by the HVO^43,44^, are also reflected in Δ*η* (denoted as smaller arrows in Fig. 3a). Another noteworthy aspect is the persistent long-term increase in Δ*η* over the five-year analysis period, at a rate of  ~ 0.25 *rad* per year (Fig. 3a). This gradual increase in Δ*η* correlates with steady ground inflation observed in the GPS and tiltmeter data, supporting a coupled process between the seaward motion of the south flank (denoted by blue arrows in Fig. 1a) and persistent magma pressurization and intrusion in the Rift Zone^35^.

### Geometric phase response to wavefield perturbations

**Fig. 4:**
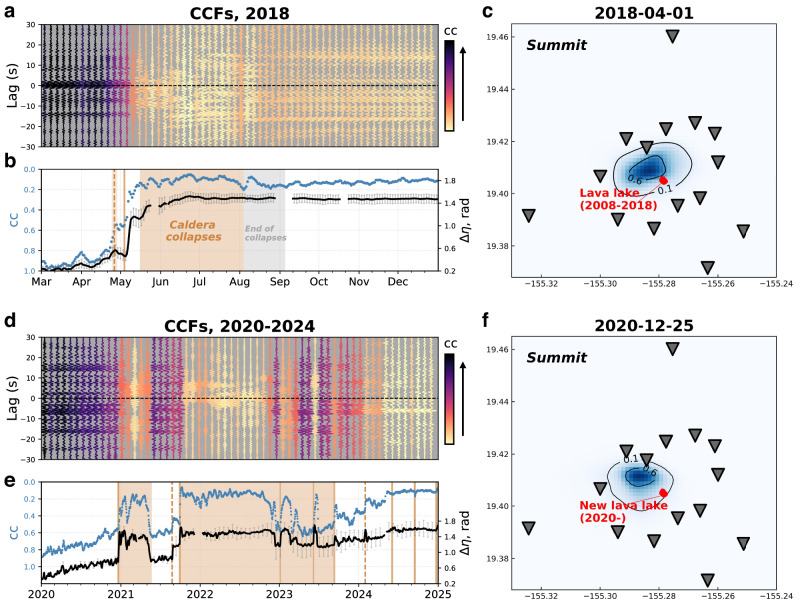
Coupling between geometric phase (Δ*η*) and seismic wavefield. **a** Temporal cross-correlation functions (CCFs) for station pair CPKD-PUHI in 2018, color-coded by their correlation coefficients relative to the averaged CCF for January 2018. **b** Comparison of the time series of CCF coherence (in blue) and Δ*η* (in black). The Δ*η* time series and eruption chronology are consistent with those in Fig. 2. Note that the CCF coherence levels are plotted inversely. **c** Localization of noise source energy. The lava lake location is approximated within HC and does not represent real-time lake boundaries. **d**–**f** are similar plots to (**a**–**c**), but for the analysis period of 2020–2024, with CCF coherence calculated relative to the averaged CCF for January 2019. All displayed CCFs and those used for energy localization are bandpass-filtered in 0.5–1.2 Hz.

Having shown that geometric phase observable (Δ*η*) evolves closely with volcanic dynamics, we next examine its relation to the wavefield reconstructed from cross-correlations, revealing its rapid and coherent response. Temporal CCFs and their coherence levels for an example pair (CPKD-PUHI) are presented to compare with the Δ*η* evolution. During the pre- and inter-eruptive periods of 2018 and 2020–2024, CCFs remain remarkably stable (Fig. 4a, d), indicating that Δ*η* and the wavefield both evolve gradually under subsurface pressurization. Once eruptions begin, transient perturbations in Δ*η* reflect strong disturbances in the wavefield by closely tracking the reductions in CCF coherence (Fig. 4b, e). Although Δ*η* is empirically correlated with waveform coherence due to their shared dependence on cross-correlation evolution, it remains conceptually and mathematically distinct from conventional coherence-based observables. To further clarify the distinction between Δ*η* and waveform coherence, we conducted a controlled spike-noise test (Supplementary Fig. S2). Temporal CCFs are randomly perturbed using two levels of impulsive noise, producing a substantial coherence degradation, with ~20% reduction in eruption-background signal separation. In contrast, the Δ*η* time series preserves the systematic eruptive evolution with nearly unchanged separation. Quantitatively, the residuals of Δ*η* are 1–2 orders of magnitude smaller than those of coherence under both noise levels, with only minor baseline shifts. This differential sensitivity demonstrates that Δ*η* does not simply track coherence loss, but remains much less affected localized waveform corruption while capturing the global geometric evolution of the wavefield.

The coherence reductions coincide with bursts of broadband energy in the spectrograms across all eruptive periods (Figs. 2 and 3a), suggesting contributions from temporary noise-source variability, including tremor activity, explosions, or lava flow. Post-eruptive recovery of both Δ*η* and CCF coherence from 2020 to 2024 (Fig. 4d, e) demonstrates wavefield re-stabilization once eruptions conclude. Following the end of 2018 eruption, Δ*η* remains elevated while CCF stabilizes with a different pattern from pre-eruption (Fig. 4a, b), likely reflecting the medium reconfiguration induced by large-scale structural changes. Supplementary Fig. S3 presents temporal CCFs for four additional station pairs, confirming the consistent co-evolution of Δ*η* and wavefield perturbations.

To further assess the stability of the ambient noise field, we localize the spatiotemporal distribution of noise-source energy (see workflow in “Methods”). During the pre-eruptive 2018 period, energy is concentrated within the summit (Fig. 4c), consistent with persistent volcanic tremor sources associated with lava lake (2008–2018) degassing and spattering^14,45–47^. After rapid lava lake draining following eruption, energy shifts outside the summit, while the formation of a new lava lake during 2020–2021 relocates energy back to the summit (Fig. 4f). The new lava lake (2020-) has been intermittently present within HC at the summit, persisting through multiple eruptive activities and enduring into late 2024. The full evolution of noise-source energy and example waveforms are shown in Supplementary Fig. S4. These results suggest that the pre-eruptive noise field is largely governed by stable volcanic tremor sources, in which Δ*η* evolves consistent with gradual subsurface magma pressurization and intrusion. Notably, even during eruptive periods characterized by pronounced noise field instability, distinct Δ*η* signatures, such as the 2018 summit-collapse sequence and the 2020–2024 post-eruptive recovery, remain clearly identifiable. To quantitatively evaluate the respective contributions of noise-source variability and subsurface medium changes, we next perform controlled sensitivity analyses in which source and medium properties are independently perturbed.

### Assessing geometric phase sensitivity to noise source variability

To constrain the potential contribution of noise-source variability to Δ*η*, we conducted controlled simulations with fixed medium properties. Ten models were constructed, comprising two source-perturbation regimes analogous to the pre-eruptive and co-eruptive periods in 2018 (Table 1). In Models 1–5, a dominant source cluster associated with lava lake activity was preserved, while 100 background sources were randomly redistributed with lower amplitudes. These simulations yield Δ*η* values of ~0.02–0.03 (mean 0.022 ± 0.003 rad), approximately one order of magnitude smaller than the observed pre-eruptive Δ*η* levels. In Models 6–10, all sources were fully randomized spatially, and source amplitudes were varied across four orders of magnitude. These extreme scenarios (Models 6–10) produce Δ*η* values of~ 1.5–1.6 (mean 1.571 ± 0.024 rad), comparable to the observed co-eruptive peak amplitudes (Fig. 2). Notably, the limited variation among Models 6–10 indicates that Δ*η* is relatively insensitive to source intensity fluctuations alone and responds primarily to large-scale geometric redistribution of sources. Detailed model configurations, representative CCFs, and Δ*η* results are provided in “Methods” and Supplementary Fig. S5.

**Table 1:** Sensitivity of Δ*η* to noise source variability

Models	Medium	Source geometry	Source amplitude^a^	Test objective	Δ*η*_*syn*_ (*rad*)
1–5	Fixed	Small perturbation around dominant source	Dominant (10^12^) + background (10^10^)	Moderate source variability (e.g., pre-eruption)	0.022 ± 0.003
6–10	Fixed	Fully randomized	10^11^–10^15^	Strong source variability (e.g., co-eruption)	1.571 ± 0.024

^a^Source amplitude represents relative scaling factors in SPECFEM2D.

These simulations provide quantitative bounds on the contribution of source variability. Moderate source perturbations do not reproduce observation-level Δ*η* signals, thereby supporting the interpretation that the pre-eruptive Δ*η* evolution primarily reflects subsurface magmatic processes. Although co-eruptive-level Δ*η* amplitudes can be reproduced under extreme and unconstrained source perturbations, they do not explain the structured temporal evolution of Δ*η* observed during the 2018 eruptive transitions and collapse sequence. During eruptive periods, elevated seismicity and tremor activity make the ambient noise field difficult to constrain, while eruptive processes further simultaneously modify both wave excitation (e.g., tremor activation, conduit resonance) and medium properties (e.g., fracturing and stress redistribution). This intrinsic source-medium coupling complicates a strict quantitative separation of their respective contributions, indicating that the co-eruptive Δ*η* evolutions reflect an integrated response of the evolving volcanic system.

### Assessing geometric phase sensitivity to subsurface medium changes

Having explored the potential contribution of source variability, we next examine the sensitivity of Δ*η* to subsurface medium changes. To isolate medium effects, we conducted independent simulations with a fixed source configuration to evaluate whether realistic magmatic perturbations produce Δ*η* evolution consistent with observations. We focus on April 18–May 3, 2018 (denoted in Fig. 2), when the summit medium responded elastically to magma pressurization^34^ and the ambient noise field remained relatively stable prior to the major eruption onset. During this period, the summit lava lake (2008–2018), directly fed by the HC reservoir, serves as a practical proxy for magma pressure perturbations through its height variations^34,35,48,49^. Accordingly, we employ pressure-driven seismic velocity changes in the simulation model, generating synthetic seismograms and computing Δ*η* to evaluate its response to these perturbations. This framework is not intended as a forward derivation of Δ*η*, but rather as a physically consistent test of whether realistic elastic perturbations produce coherent Δ*η* evolution. This simulation strategy provides two advantages. First, magma pressure-driven elastic responses at Kı̄lauea are well documented, where compression closes cracks, stiffens the rock frame, and increases seismic velocity^14–16^. This pressure-velocity relationship can be quantified using a poroelastic solution for volumetric strain, which has been widely applied under diverse geological conditions^50–53^. Second, this framework allows direct comparison between the sensitivities of Δ*η* and seismic wavespeed to identical pressure perturbations. We verify this poroelastic strain solution^50^ and constrain parameters by simulating vertical surface displacements and comparing them with GPS observations (Supplementary Fig. S6). Their close agreement supports the applicability of this solution for characterizing the medium response. Details of the poroelastic formulation are provided in “Methods”.

**Fig. 5:**
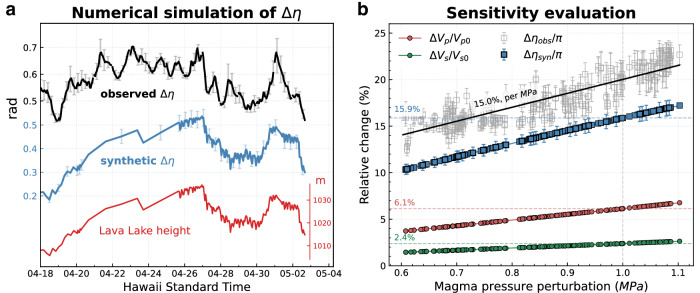
Sensitivity of geometric phase to medium properties changes. **a** Comparison of variations in observed Δ*η*, synthetic Δ*η*, and the summit lava lake height. The simulated period is indicated in Fig. 2. Both the observed and synthetic Δ*η* represent the mean level across four consistent stations, while the error bars denote their standard deviation. **b** Relative changes in Δ*η*, as well as compressional (*V*_*p*_) and shear (*V*_*s*_) wavespeeds, in response to magma pressure perturbations. The observed Δ*η* shows a fitted change rate of 15.0% per MPa (thick black line). Wavespeed changes are averaged from the grids of four virtual receivers at the surface (Supplementary Fig. S13b).

Figure 5a shows the synthetic Δ*η* derived from the numerical simulation using the input lava lake height variations. The systematic 0.2–0.3 offset between observed and synthetic Δ*η* is expected given the simplified nature of the simulation model, including idealized ambient noise sources, pressure-velocity coupling, a 1-D background structure, and the magmatic pressure approximation. Despite these simplifications, the synthetic results reproduce the first-order temporal evolution of Δ*η*, supporting the physical interpretability of the geometric phase sensing. In Fig. 5b, we quantify the sensitivity of Δ*η* to the imposed magma pressure perturbations. For direct comparison, Δ*η* is normalized by *π* as $\frac{\Delta \eta }{\pi }\times 100\%$, representing its full theoretical range, while wavespeed are expressed as relative percentage changes ($\frac{\Delta V}{{V}_{0}}\times 100\%$). The synthetic Δ*η* exhibits a nearly linear response due to the imposed linear pressure-velocity relationship in the strain solution. The observed Δ*η* shows a fitted slope of 15.0% per MPa, in close agreement with the 15.9% change predicted in the synthetic Δ*η* under equivalent pressure perturbations (see details of the slope fitting in Supplementary Fig. S7). The 15.9% change in Δ*η* is substantially larger than the corresponding 6.1 and 2.4% changes in compressional (*V*_*p*_) and in shear (*V*_*s*_) wavespeeds, respectively, indicating greater sensitivity of geometric phase sensing to subsurface magma pressurization. Overall, these simulations support that Δ*η* is highly sensitive to pressure-induced changes in the elastic medium, supporting its use as an indicator of gradual and subtle precursory magmatic unrest.

## Discussion

By leveraging geometric phase sensing from ambient seismic noise, we capture the full evolution of volcanic dynamics at Kı̄lauea with unprecedented sensitivity and stability. The strength of this approach lies in extracting the global geometric evolution of the wavefield from a subset of stations and condensing the full-wavefield response into a single observable. This provides a geometry-based perspective, rooted in topological acoustics, for characterizing medium variations.

Compared with previous seismic monitoring studies at Kı̄lauea, geometric phase sensing offers three practical advantages: (1) Performance under strongly varying noise-source conditions. Δ*η* continues to exhibit coherent and physically meaningful eruptive-related signatures even during periods of reduced waveform coherence. It captures the co-eruptive state transitions and the 2018 summit collapse sequence, as well as the multiple onsets and terminations of eruption episodes in 2020–2024. These structured temporal patterns can be difficult to resolve in conventional *dv*/*v* measurements that rely on waveform coherence. Our reproduction of *dv*/*v* (Supplementary Text S1) yields less interpretable and more variable signals across station pairs, indicating stronger disruption by evolving noise-source conditions (Supplementary Fig. S8). A few studies addressed these challenges using repeating-earthquake doublets^17^ or dense geophone arrays^16^, which require additional data constraints. We note that the adopted *dv*/*v* estimation here represents a conventional baseline method, more advanced frequency-time or reference-independent procedures^54^ may better accommodate heterogeneous or evolving medium. In this strongly coupled volcanic environment, however, Δ*η* preserves coherent eruptive signatures without specialized stabilization procedures, suggesting its practical value for monitoring where source and medium processes evolve simultaneously. (2) Temporally coherent tracking of magmatic pressurization prior to eruption. For instance, the April 26, 2018 ground inflation peak (Day −4 in Fig. 2) was rarely captured in previous *dv*/*v* studies, likely due to complex pressure accumulation and reduced bulk edifice strength^15,19^. A comparable inflation signal was reported before, in which the associated *dv*/*v* peak occurred around April 24 for caldera stations and was further delayed at stations located eastward^20^. We also examine a similar peak mismatch in our own *dv*/*v* reproduction, with a  ~2-day lag (inset of Supplementary Fig. S8a). In contrast, the temporal evolution of Δ*η* provides a more coherent indicator that closely aligns with GPS and lava lake observations (Fig. 2), highlighting its value in identifying eruptive precursors while reducing interpretational ambiguity. (3) Computational efficiency. Geometric phase sensing requires no time-window selection; full CCF traces are directly incorporated into the state vector to sample wavefield geometry. The Δ*η* calculation itself involves only fast Fourier transforms and vector dot products, enabling fast, automated processing. Excluding cross-correlations, processing all daily CCFs into Δ*η* takes ~ 2 min for 15 stations in 2018 and ~3 min for 7 stations in 2020–2024 on a single Intel Xeon w3-2423 CPU. This Δ*η* calculation can be fully reproduced with an integrated script in our code package, making it well-suited for near-real-time monitoring.

Methodological choices also shape the implementation of the geometric phase sensing. The first is the reference time period, which defines the reference state vector **C**_*ref*_ (in Step 3 of Fig. 1c) and serves as the baseline for subsequent wavefield evolution. Because Δ*η* is defined as the angular evolution of the state vector relative to this baseline, Δ*η* is inherently measured with respect to the chosen reference condition. In this study, we empirically define a one-month pre-eruptive background stage and average state vectors over this period as the **C**_*ref*_. This approach prevents Δ*η* from artificially reaching its lower bound of zero when a single time step is used. The selection of the reference period is therefore empirical and should be adapted to the specific monitoring context and application. The second reference setup is the reference station used for wavefield sampling (in Step 2 of Fig. 1c). Tests across all possible reference stations show highly consistent temporal trends of Δ*η*, with modest amplitude variability (Supplementary Figs. S9b and S10b). We therefore present the mean Δ*η* along with its standard deviation. This variability is partly influenced by the number of available stations (15 for 2018 and 7 for 2020–2024). Frequency dependence also influences interpretation. We present the time-frequency functions of Δ*η* and test sub-bands in the range of 0.4–1.6 Hz and 0.8–1.6 Hz for two analysis periods (Supplementary Figs. S9a and S10a). The small variations in Cohen’s *D* metrics across these frequency ranges suggest comparably robust separation between eruptive and background signals. Lower-frequency bands dominated by oceanic microseisms (≤0.3 Hz) show smaller Δ*η* amplitudes and weaker eruptive responses, reflecting their longer wavelengths and reduced sensitivity to shallow, localized magma-related structural changes. Accordingly, we average Δ*η* time series over 0.6–1.0 Hz (for 2018) and 1.0–1.2 Hz (for 2020–2024) to balance signal strength and temporal stability. These frequency ranges align with previous ambient noise studies at Kı̄lauea summit^14,15,18,19^ and probe a central depth of ~ 1 km below surface^14,15,19^. Importantly, the geometric phase framework naturally provides a time-frequency representation, enabling robust identification along the full frequency range without prior selection.

The current geometric phase formulation reflects the global, wavepath-integrated nature of Δ*η*, which limits the direct derivation of spatial sensitivity kernels comparable to parameter-specific inversion quantities such as *dv*/*v*. Accordingly, this framework emphasizes robust temporal tracking rather than explicit spatial inversion. To explore the spatial interpretability of Δ*η*, we conducted a targeted subarray-level analysis (Supplementary Fig. S11). Subarrays constructed from southern station group systematically exhibit higher Δ*η* amplitudes than those from northern group at the inflation peak on April 26, 2018, consistent with independent GPS observations and interpretations of southern intrusive processes. These subarray-level results should be interpreted as qualitative indications of potential spatial sensitivity rather than a quantitative spatial inversion or localization of subsurface perturbations. Nevertheless, the observed variations suggest that Δ*η* may retain spatially informative behavior when evaluated using carefully designed station subsets, highlighting a potential pathway toward spatially resolved geometric phase sensing.

We note that the poroelastic strain solution used to describe the pressure-driven medium response does not fully account for previously observed velocity changes, which likely involve additional complex mechanisms^15,19^. Nonetheless, it provides a simplified and practical framework that solves the first-order relation between Δ*η* and magma pressure perturbations. We constrain most parameter values either from literature or our estimations (Supplementary Table S1). Although some values may vary over time, the constant values adopted are representative of the solution. Future work incorporating more precise parameter values and explicit medium processes could further enhance the quantification of subsurface changes and strengthen validation of the geometric phase observations.

In summary, we demonstrate that the geometric phase observable (Δ*η*) extracted from ambient seismic noise provides a sensitive measure of volcanic dynamics. At Kı̄lauea, Δ*η* continuously resolves precursory magma pressurization, co-eruptive fractural caldera collapse, and post-eruptive recovery, offering a comprehensive view of processes that can be difficult to capture in previous studies. By introducing an intrinsic geometric perspective of the seismic wavefield, this approach moves beyond pairwise waveform measurements and establishes an applied solution for tracking wavepath-integrated subsurface perturbations. Leveraging abundant open-source seismic data, the workflow enables continuous, near-real-time monitoring of volcanic systems worldwide. Importantly, the method draws on principles of topological acoustics, opening avenues for interpreting wavefield evolution through geometric and topological features.

Looking ahead, we envision geometric phase sensing as a generalized framework for probing seismic wavefield evolution in dynamic environments. Because Δ*η* encodes global wavefield changes, the approach is applicable beyond volcanology to settings where subtle structural variations influence seismic propagation. Potential applications include monitoring ice-sheet and subglacial dynamics; groundwater and streamflow fluctuations; permafrost degradation associated with climate change; and fault zone evolution driven by tectonic or magmatic processes. Overall, geometric phase sensing may support environmental and geohazard monitoring by providing a wavefield-based measure of subsurface changes; future efforts should integrate Δ*η* with complementary datasets and evaluate its performance across diverse observational settings.

## Methods

### Seismic noise recordings and cross-correlations

We select a total of 15 open-source broadband seismometers from the HVO Network (network code: HV)^55^, all with a sampling rate of 100 Hz, and collect their vertical component recordings. The data coverage for each station is displayed in Supplementary Fig. S12. We apply the open-source MSNoise package^56^ to preprocess seismic recordings and perform cross-correlations (Step 1 in Fig. 1c). Seismic noise recordings are preprocessed by removing instrumental response, demeaning, and detrending. A wide bandpass filter of 0.01–49.9 Hz is applied, and signals exceeding three times the root-mean-square amplitude are removed as outliers. We then slice the preprocessed seismograms into 1800-s windows, cross-correlate them between different stations, and stack the results every day to produce the temporal (daily) cross-correlation functions, *CCF*(*t*). We further apply a 5-day moving average to enhance the data quality of *CCF*(*t*). For the two primary long-term Δ*η* analyses (Δ*η* in Figs. 2 and 3a), we set the investigation time step *T* as one day by using 5-day moving-averaged temporal *CCF*_*T*_(*t*). For the three short-term analyses (inset panel of Fig. 2, observed Δ*η* in Fig. 5a and Supplementary Fig. S1b), we directly cross-correlate one-hour-long noise windows and produce hourly *CCF*_*T*_, while the preprocess steps remain the same. We test CCF stacking windows of 1, 3, and 6 h and find consistent short-term Δ*η* patterns (Supplementary Fig. S1c). We therefore directly use hourly *CCF*_*T*_ as a balance between noise reduction and temporal resolution.

### Building the state vector and measuring geometric phase

The temporal *CCF*_*T*_ is used to sample the geometry of a wavefield and build the state vector (Step 2 in Fig. 1c). To sample the valid geometry of a wavefield, we select a reference station and include only its cross-correlations with other stations. The resulting state vector, **C**_*ω*,*T*_ captures both spectral and temporal characteristics of the sampled wavefield by including the spectral *CCF*_*T*_ at investigation time step *T*. We express **C**_*ω*,*T*_ as:

1 $${{{{\bf{C}}}}}_{\omega,T}=\frac{1}{\sqrt{{A}_{1}^{2}+{A}_{2}^{2}+{A}_{3}^{2}+...+{A}_{n}^{2}}}\left(\begin{array}{c}{A}_{1}{e}^{i{\phi }_{1}}\\ {A}_{2}{e}^{i{\phi }_{2}}\\ {A}_{3}{e}^{i{\phi }_{3}}\\ ...\\ {A}_{n}{e}^{i{\phi }_{n}}\end{array}\right)\,,$$

where **C**_*ω*,*T*_ consists of *N* components, indexed by *n*, consistent with the station pairs *N* in Fig. 1c. In each component, ${A}_{n}{e}^{i{\phi }_{n}}$ represents the spectral *CCF*_*T*_ obtained after applying the fast Fourier transform, enabling **C**_*ω*,*T*_ to capture wavefield information across different frequencies *ω*. The denominator normalizes over all *N* components by integrating their magnitudes, *A*_*n*_, including both real and imaginary parts. Unlike conventional array processing, which simply aggregates all station pairs, this state vector samples a geometric (vectorial) representation of the wavefield by linking a reference station to each other. This unique setup is the key concept of the geometric phase approach, and we can sample independent wavefields by changing the reference station.

Each component of the resulting state vector can be projected along the axes of an abstract Hilbert space with corresponding *N* dimensions (Step 3 in Fig. 1c). We then employ the geometric phase change Δ*η*(*ω*, *T*) to describe the evolution of the wavefield through tracking the rotated angle of two state vectors **C**_*ω*,*T*_ in the same Hilbert space:

2 $$\Delta \eta (\omega,T)=arccos\left[{{{\bf{Re}}}}\left({{{{\bf{C}}}}}_{\omega,ref}^{*}\cdot {{{{\bf{C}}}}}_{\omega,T}\right)\right]\,and\,\Delta \eta \in [0,\pi ]\,.$$

**C**_*ω*,*ref*_ represents a reference state vector defining the baseline for measuring the rotated angle of **C**_*ω*,*T*_. The *arccos* function is applied to the real part (**Re**) of their dot product, and ^*^ denotes the complex conjugate. Therefore, the direct output of Δ*η*(*ω*, *T*) is a time-frequency function, from which a time series of Δ*η*(*T*) can be extracted over a selected frequency range (output from Step 3 in Fig. 1c).

We separately applied the workflow to 15 stations for the 2018 analysis and 7 stations for the 2020–2024 analysis, as 8 stations lacked data during key eruptive periods (see data coverage in Supplementary Fig. S12). For the 2018 analysis (including the short-term co-eruptive evolution in Supplementary Fig. S1), the reference state vector was defined as the average of temporal state vectors within January 2018, and Δ*η* time series was averaged over the 0.6–1.0 Hz frequency band (Figs. 2 and 5a). For the 2020–2024 analysis, the reference state vector was defined within January 2020, and Δ*η* time series was averaged over the 1.0–1.2 Hz (Fig. 3a). In “Discussion”, we discuss more details about the methodological setup in the geometric phase measurements.

### Noise source energy localization

We apply the Matched Field Processing (MFP) method^57,58^ to localize noise source energy, which propagates the energy of primary wave arrivals in the CCF to all possible source locations in space. We calculate the differential traveltime Δ*t*_*ij*_ from stations *i* and *j* to a point in the 2-D source space:

3 $$\Delta {t}_{ij}=\frac{{d}_{i}-{d}_{j}}{{v}_{0}}\,,$$

where *d*_*i*_ denotes the distance from the point to station *i*, and *d*_*j*_ is for station *j*. We use a constant velocity *v*_0_ of 1.0 km/s based on the primary wave arrivals in CCF (denoted in Supplementary Fig. S4a). Next, we stack the energy of CCFs from all station pairs and propagate it to the source space according to the calculated Δ*t*_*ij*_. Here, we use the amplitude of squared envelope function *S*(*t*) for each CCF (see an example in Supplementary Fig. S4b). The MFP power map *P*(*x*, *y*) represents the stacked energy from *S*(*t*) of all station pairs at each source point (*x*, *y*) in the 2-D space Ω:

4 $$P(x,y)={\prod }_{n=1}^{N}{\int }_{\Delta {t}_{ij}-\tau }^{\Delta {t}_{ij}+\tau }{S}_{n}(t)dt,\,(x,y)\in \Omega \,.$$

After these steps, we project the energy within a time window of the squared envelope functions *S*(*n*) for a total *N* station pairs. The window is defined by the traveltime Δ*t*_*ij*_, extending a range of *τ* = ± 5 s. We multiply the MFP power maps across station pairs to suppress imaging artifacts.

### Poroelastic solution for volumetric strain

We model the shallow summit medium as an elastic porous rock, where pressure perturbations from the underlying magma reservoir drive elastic strain through pore pressure changes. Accordingly, we approximate the pore pressure variations Δ*P*_*p*_(*t*) by the magma pressure variations Δ*P*_*m*_(*t*):

5 $$\Delta {P}_{p}(t)\approx \Delta {P}_{m}(t)\approx {\rho }_{m}\cdot g\cdot \Delta {h}_{m}(t)\,,$$

where Δ*h*_*m*_(*t*) represents the lava lake height changes, with a baseline of 992.5 m at 2018-01-01, 05:00:00 (assigned for the reference model in the Δ*η* simulation). *ρ*_*m*_ is the magma density, fixed at 2550 kg/m^3^ within the estimated range of 2550–2650 kg/m^3^ for the HC reservoir^34^, although density likely increases with depth due to compression.

We adapt the poroelastic strain solution^50^ into our setup, yielding the strain amplitude *A*(*t*) as:

6 $$A(t)=\frac{(1+\nu )(1-2\nu )}{(1-\nu )E}k{\alpha }_{Biot}\Delta {P}_{p}(t)\sqrt{\frac{\kappa }{\omega }}{e}^{\frac{\pi }{4}}\,,$$

where *α*_*Biot*_ is the Biot’s coefficient describing the contribution of pore pressure to effective stress, *κ* is the hydraulic diffusivity that governs how rapidly pressure perturbations propagate. *ν* and *E* are the Poisson’s ratio and Young’s modulus of the rock, *k* and *ω* are the horizontal wavenumber and angular frequency of the pressure perturbations.

The resulting strain *A*(*t*) is converted into compressional and shear wavespeed changes (Δ*V*_*p*_ and Δ*V*_*s*_) and vertical surface displacement. Derivations are given in Supplementary Text S2, and all parameter values with references are listed in Supplementary Table S1.

### Numerical simulation of the geometric phase

We use the spectral-element solver SPECFEM2D (Version 8.1.0)^59^ to simulate wave propagation across a two-dimensional elastic medium and then calculate synthetic Δ*η* variations. The computational domain was discretized into a 120 × 20 mesh with each element size of 100 m (Supplementary Fig. S13a). The effective point spacing is Δ*x* ≈ 25 m, owing to the inclusion of 5 internal points per element. Given the empirical safety factor *n* = 8 and the minimum shear wavespeed of *V*_*min*_ = 1500 m/s, we calculate the maximum frequency as ${f}_{max}\approx \frac{{V}_{min}}{n\Delta x}=7.5$ Hz. This mesh configuration allows us to simulate accurate waveforms up to 7.5 Hz, effectively covering our frequency range of interest from 0.6 to 1.0 Hz. A free surface boundary condition is applied at the top edge, while absorbing boundary layers are implemented on the lateral and bottom edges to minimize artificial reflections.

To approximate the ambient seismic noise field in the Kı̄lauea volcano area, we generate wavefields from a total of 500 pressure point sources distributed across the model space. We first construct a reference model: 400 sources are placed within the half-buried reservoir and assigned amplitude factors of 10^12^ to represent dominant volcanic tremor sources, while the remaining 100 sources are randomly distributed across the model space with amplitude factors of 10^10^ (Supplementary Fig. S13b). Each source is excited with a random time shift within a 120-s window, and the source time function is modeled as a Ricker wavelet with a center frequency randomly ranging from 0.1 to 1 Hz. The initial velocity structure^60^ (Supplementary Fig. S14a) is assigned to the reference model. We deployed four virtual stations spanning a horizontal distance of 12 km within the model domain, matching their discrete linear geometry in field (Supplementary Fig. S13c). This reference model serves as the baseline for calculating synthetic Δ*η* in sensitivity analyses of both source variability (Table 1) and subsurface medium change (Fig. 5).

For the source variability tests (Table 1), Models 1–5 perturb only 100 background sources with different randomized spatial distributions, while keeping the 400 dominant sources fixed. In Models 6–10, all 500 sources are fully perturbed with different random spatial distributions and progressively increasing amplitude scales (from 10^11^ to 10^15^). For all ten source variability models, medium properties are kept fixed by applying the same velocity structure as the reference model. In contrast, for the subsurface medium change tests (Fig. 5), we perturb velocity structure according to the computed wavespeed changes, while keeping the source configuration consistent with the reference model. For all modeling cases above, a 125-s noise seismogram is generated at each station (see an example in Supplementary Fig. S14b). The vertical-component seismograms are preprocessed, sliced into 20-s windows, and cross-correlated between station pairs. The resulting functions are then stacked to obtain synthetic CCFs (see an example in Supplementary Fig. S14c). Synthetic Δ*η* are then computed using identical processing parameters as in the observations: the same four stations are used, and results are averaged over 0.6–1.0 Hz.

## Supplementary information

Supplementary Information

Transparent Peer Review file

## Data Availability

The continuous seismic recordings (network code: HV)^55^ used in this study are open and available at the EarthScope Data Management Center (http://service.iris.edu/fdsnws/dataselect/1/). Ambient noise cross-correlations were computed using the open-source MSNoise package^56^. GPS data for station CRIM were sourced from UNAVCO^61^ and we collected the second-processed time series from the Nevada Geodetic Laboratory (https://geodesy.unr.edu/NGLStationPages/stations/CRIM.sta)^62^. Ground tilt^63,64^ and 2008–2018 lava lake height recordings^65,66^ data were obtained from USGS ScienceBase (https://www.sciencebase.gov/catalog/). Earthquake catalogs were collected from a published study^67^ and the USGS ComCat system (https://earthquake.usgs.gov/earthquakes/search/). Eruption chronology was referenced from the Smithsonian Institution Global Volcanism Program database (https://volcano.si.edu/) and a published study^35^. Simulations of geometric phase were conducted using SPECFEM2D (Version 8.1.0)^59^. All figures were plotted using Generic Mapping Tools^68^ and Matplotlib libraries^69^.

## Code availability

Code used for the major analyses in this study is openly available at both GitHub (https://github.com/Badboy-Luo/geometric-phase-kilauea) and Zenodo^70^. This code package includes our custom routines for geometric phase (Δ*η*) calculation and match field processing (MFP) tests, and parameter files for the MSNoise and SPECFEM2D to reproduce seismic cross-correlation and numerical simulations of Δ*η*.
