# Supplementary material for: Geometric phase sensing using seismic waves for comprehensive volcano monitoring at Kı̄lauea Hawaii
Source: Nat Commun. 2026 Jun 26;17:7988. doi: 10.1038/s41467-026-73998-x (PMC13448816; doi:10.1038/s41467-026-73998-x)
Supplement: Supplementary file 1 [file 41467_2026_73998_MOESM1_ESM.pdf]

Supplementary Materials for

"Geometric phase sensing using seismic waves for comprehensive volcano monitoring at Kīlauea Hawaii"

Bingxu Luo * [1,2], Susan Beck[1,2], Pierre Deymier[1,3], Keith Runge[1,3], Eric Kiser[2], Pranabendu Moitra[2], Falk Huettmann[1,4], Samy Missoum[1,5], Marat Latypov[1,3], Elizabeth Whitney[1,4], Jiayang Wang[1,3], and Sinan S. Schabib[1,5]

[1]*New Frontiers of Sound Science and Technology Center, The University of Arizona, Tucson, USA*

[2]*Department of Geosciences, The University of Arizona, Tucson, USA*

[3]*Department of Materials Science and Engineering, The University of Arizona, Tucson, USA*

[4]*-EWHALE Lab-. Department of Biology and Wildlife. Institute of Arctic Biology, University of Alaska Fairbanks, Fairbanks, USA*

[5]*Department of Aerospace and Mechanical Engineering, The University of Arizona, Tucson, USA*

This PDF file includes:

Supplementary Texts S1 and S2

Supplementary Table S1

Supplementary Figures S1-S14

References

*Corresponding author: Bingxu Luo, bingxuluo@arizona.edu

Text S1: Relative seismic velocity change (dv/v) measurements

We followed nearly the same raw seismic data processing and cross-correlation procedure as described in "Methods" of the main text. The only modification was applying a bandpass filter of 0.5–1.2 Hz, chosen because this frequency band exhibits relatively stable spectrograms (Figures 2 and 3) and cross-correlation patterns (Figures 4a and d in the main text) during non-eruptive periods, and is consistent with analysis frequencies used in previous dv/v studies at Kīlauea. To ensure consistency between the two $\Delta\eta$ analysis periods, we retrieved daily cross-correlation functions (CCFs) using 15 stations for the 2018 analysis and 7 stations for the 2020–2024 analysis.

We measured dv/v using the Moving-Window Cross-Spectrum (MWCS) technique (Clarke et al., 2011) implemented in the MSNoise package (Lecocq et al., 2014). MWCS estimates coherent phase shifts (dt) in the spectral domain between daily and reference CCFs. These dt estimates are fitted through a weighted linear regression to obtain daily travel-time shifts (dt/t), and then inverted to derive relative velocity changes ($dv/v = -dt/t$). For the measurements, we used the early coda window defined by a velocity of 0.8 km/s (can be referenced in Figure S4a) on both the causal (positive) and acausal (negative) sides of the CCFs. The window length was set to 10 s, extending from the defined velocity arrival. The reference CCFs were constructed by stacking daily CCFs over January 2018 (for the 2018 analysis) and over October 2019 (for the 2020–2024 analysis), consistent with the two $\Delta\eta$ analyses. The resulting dv/v time series for both periods are shown in Figure S8. Detailed MWCS parameters are provided in the table below:

**Filter**	**velocity defines lag windows**	**lag window length**	**MWCS step length**	**MWCS moving step**
0.5-1.2 Hz	0.8 km/s	10 s	5 s	2 s
**Other parameters in regression of dt/t**	**minimum coherence**	**maximum error**	**maximum time shift dt**	
	0.75	0.5 s	0.5 s	

Text S2: Solving seismic wavespeed changes and surface displacement based on the poroelastic strain

Building on the poroelastic strain $A(t)$ solution described in the main text:

$$A(t) = \frac{(1+\nu)(1-2\nu)}{(1-\nu)E} k\alpha_{Biot} \Delta P_p(t) \sqrt{\frac{\kappa}{\omega}} e^{\frac{\pi}{4}} \quad , \tag{S1}$$

the corresponding changes in relative compressional (ΔV_p) and shear (ΔV_s) wavespeeds can be expressed as (Tsai, 2011):

$$\begin{aligned} \Delta V_p(t) &\approx A(t) e^{-k\cdot d} \frac{1}{\lambda+2\mu} [(2\lambda+5\mu+2m)(1-k\cdot d)+(3\lambda+5\mu+2l+2m)(1-2\nu)] \quad , \\ \Delta V_s(t) &\approx A(t) e^{-k\cdot d} \left[(1-k\cdot d)+(\frac{\lambda+3\mu+m}{\mu})(1-2\nu)\right] \quad , \end{aligned} \tag{S2}$$

in which d is the investigated distance, we calculate it as the centroid distance from each grid point to the approximated magma reservoir in the velocity model (we show an example in Figure S13a). λ and μ are the first and second Lamé parameters, l and m are the first and second Murnaghan third-order elastic constants (Murnaghan, 1937), respectively.

The hydraulic diffusivity of the host rock, κ (in Equation S1) can vary substantially across different rock types. To constrain κ, we perform a grid search by fitting simulated vertical surface displacement to GPS observations under the same strain framework. Following Tsai (2011), the vertical displacement can be expressed as:

$$u_z(t) \approx \frac{A(t)}{k} e^{-k\cdot d}(1-2\nu+k\cdot d) \quad . \tag{S3}$$

Here, we define d as the distance from the magma reservoir to GNSS station CRIM (see its location in Figure 1b in the main text). The optimal κ is determined by minimizing the mean square residual between time series of simulated u_z and observed vertical displacements at CRIM, yielding a best-fit value of 0.022 m^2/s (Figure S6).

For the remaining parameters in the poroelastic solution (Supplementary Table S1), we adopt val-

ues constrained by published references. Although some of these parameters may vary over time, we assume constant values that are representative for the implementation. The Biot coefficient (α_{Biot} in Equation S1) reflects the contribution of pore pressure to the effective stress in a porous medium, with values close to 1 indicating a highly compliant pore structure. We adopt $\alpha_{Biot} = 0.7$ as a representative value. A sensitivity test with $\alpha_{Biot} = 0.6$ yields $\Delta\eta$ values only 0.051 lower than the synthetic $\Delta\eta$ in Figure 5a in the main text, a minor offset that does not affect the temporal evolution of the results.

Table S1: Parameters used in solving poroelastic strain and seismic wavespeed

Symbol	Parameter (Unit)	Value	Reference
ν	Poisson's ratio	0.3	Tsai (2011)
μ	Host rock shear modulus (Pa)	3.08×10^9	Anderson et al. (2019)
E	Young's modulus (Pa)	$2\mu(1+\nu)$	Relation
λ	First Lamé parameter (Pa)	$2\mu\nu/(1-2\nu)$	Relation
m	Second Murnaghan parameter (Pa)	$2 \times 10^4 \cdot \mu$	Maximum approximation
l	First Murnaghan parameter (Pa)	$2 \cdot m$	Empirical relation
d	Investigated distance (m)	calculation	Figure S13a
κ	Diffusivity (m^2/s)	0.022	Estimation (Figure S6)
α_{Biot}	Biot coefficient	0.7	Tsai (2011)
k	Horizontal wavenumber (m^{-1})	$2\pi/40000$	Tsai (2011)
ω	Angular frequency (s^{-1})	2×10^{-7}	Tsai (2011)
ρ_m	Density of the magma (kg/m^3)	2550	Anderson et al. (2019)
$\Delta h_m(t)$	Height changes of the lavalake (m)	up to $\sim$44	Datasets (Patrick et al., 2019, 2021)

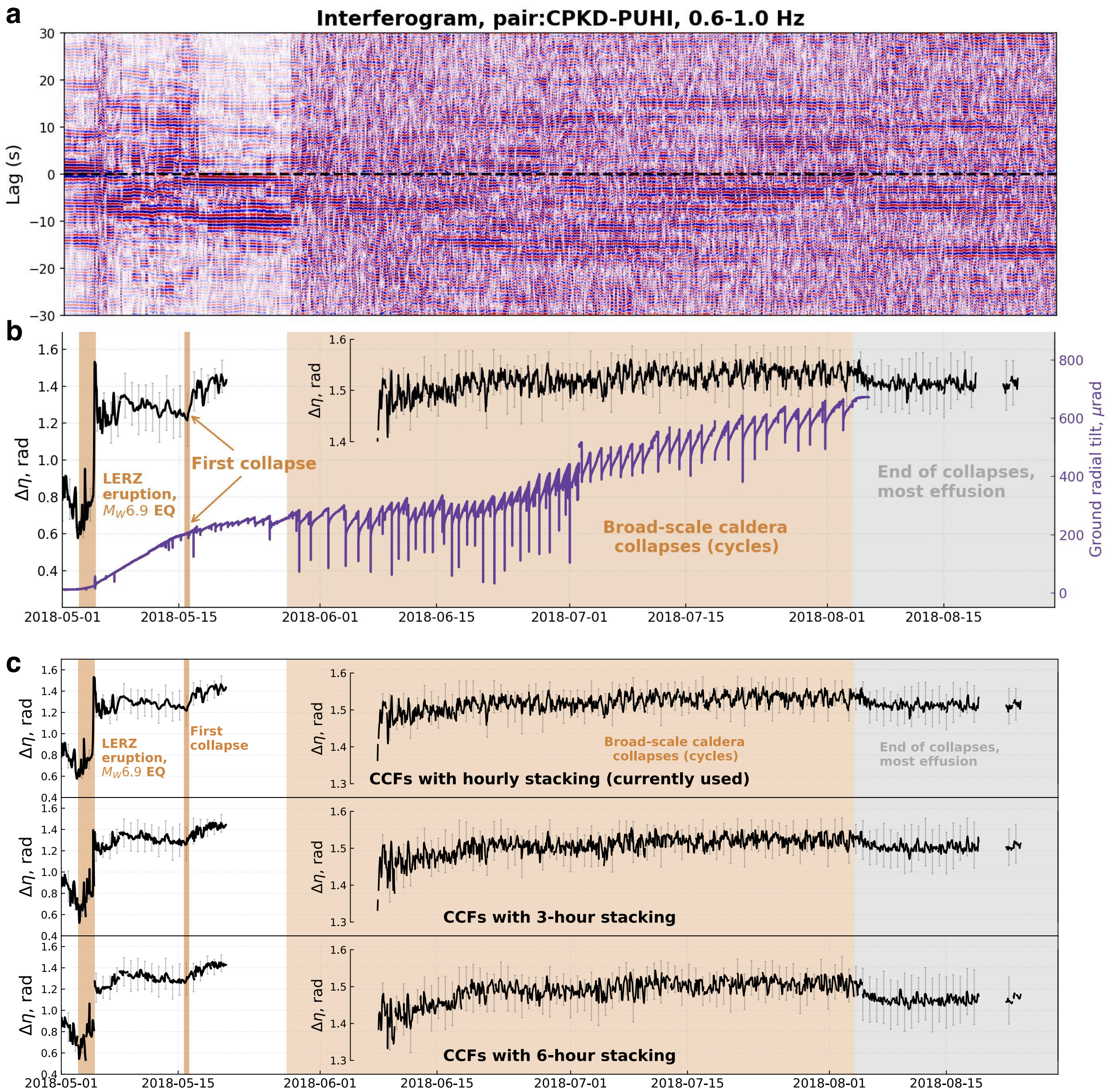

Figure S1: 2018 co-eruptive evolution of $\Delta\eta$, supporting Figure 2 in the main text. (a) An example temporal (hourly) cross-correlation functions for the station pair CPKD-PUHI. (b) Correlation between $\Delta\eta$ and ground radial tilt. Absent $\Delta\eta$ denotes data recording gaps of seismometers. (c) Comparison of $\Delta\eta$ evolution based on three CCF stacking windows (1, 3, and 6 hours). We directly use hourly CCFs for short-term $\Delta\eta$ analyses in the main text.

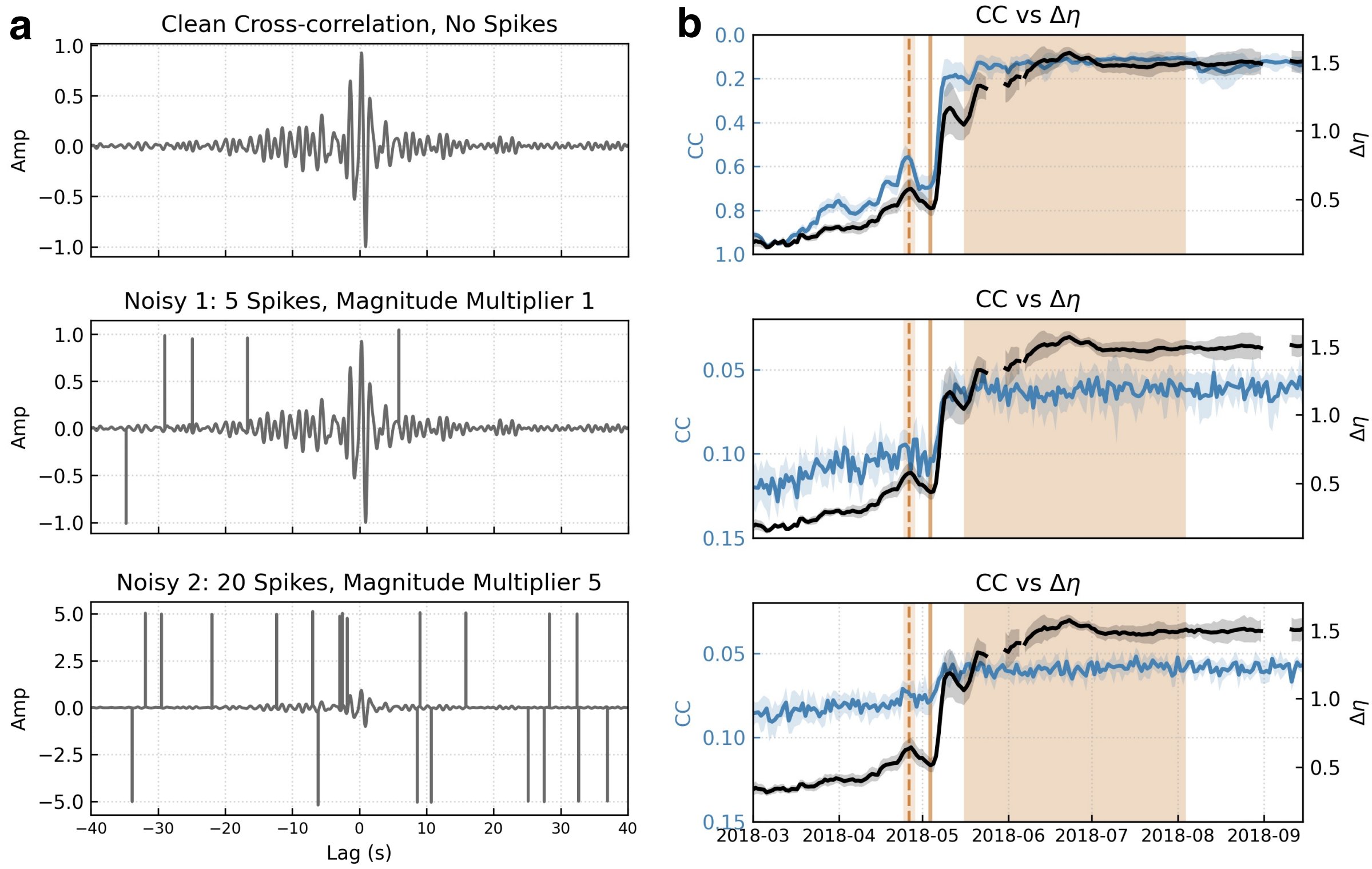

Metric	Clean (No spikes)	Noisy 1 (5 spikes + Mag 1)	Noisy 2 (20 spikes + Mag 5)	Change (%)
Cohen's D, **CC**	8.77	6.98	6.64	-20%, -24%
Cohen's D, $\Delta\boldsymbol{\eta}$	8.15	8.17	8.21	+0.2%, +0.7%
RMS, **CC**	/	0.450	0.468	/
RMS, $\Delta\boldsymbol{\eta}$	/	0.003	0.085	/
NRMS, **CC**	/	1.268	1.318	/
NRMS, $\Delta\boldsymbol{\eta}$	/	0.006	0.145	/

Figure S2: Spike-noise tests for differentiating geometric phase ($\Delta\eta$) and correlation coefficient (CC). All results are computed using consistent four stations: SBL, BYL, KKO, OBL. (a) Example cross-correlation functions: a clean reference (no noise), Noisy 1 generated by adding five spikes (amplitude = 1), and Noisy 2 generated by adding twenty spikes (amplitude = 5). Spikes are randomly inserted across all temporal cross-correlation functions. (b) Temporal evolution of CC and $\Delta\eta$ during the 2018 eruption based on the corresponding tests. Solid curves show the mean CC and $\Delta\eta$, shaded areas indicate the standard deviation across all station pairs (for CC) and across all reference-station choices (for $\Delta\eta$). Statistical metrics summarizing the responses of CC and $\Delta\eta$ are provided in the table.

Cohen's D: Quantifies the separation between eruption and background signals by following the same procedure as in Supplementary Fig. S9.
RMS: Root Mean Square error evaluates the absolute deviation between noisy and clean results (units are the same as signals).
NRMS: Normalize RMS by the data range to enable scale-independent comparison (unitless), defined as: $NRMS = \frac{RMS}{x_{max} - x_{min}}$.

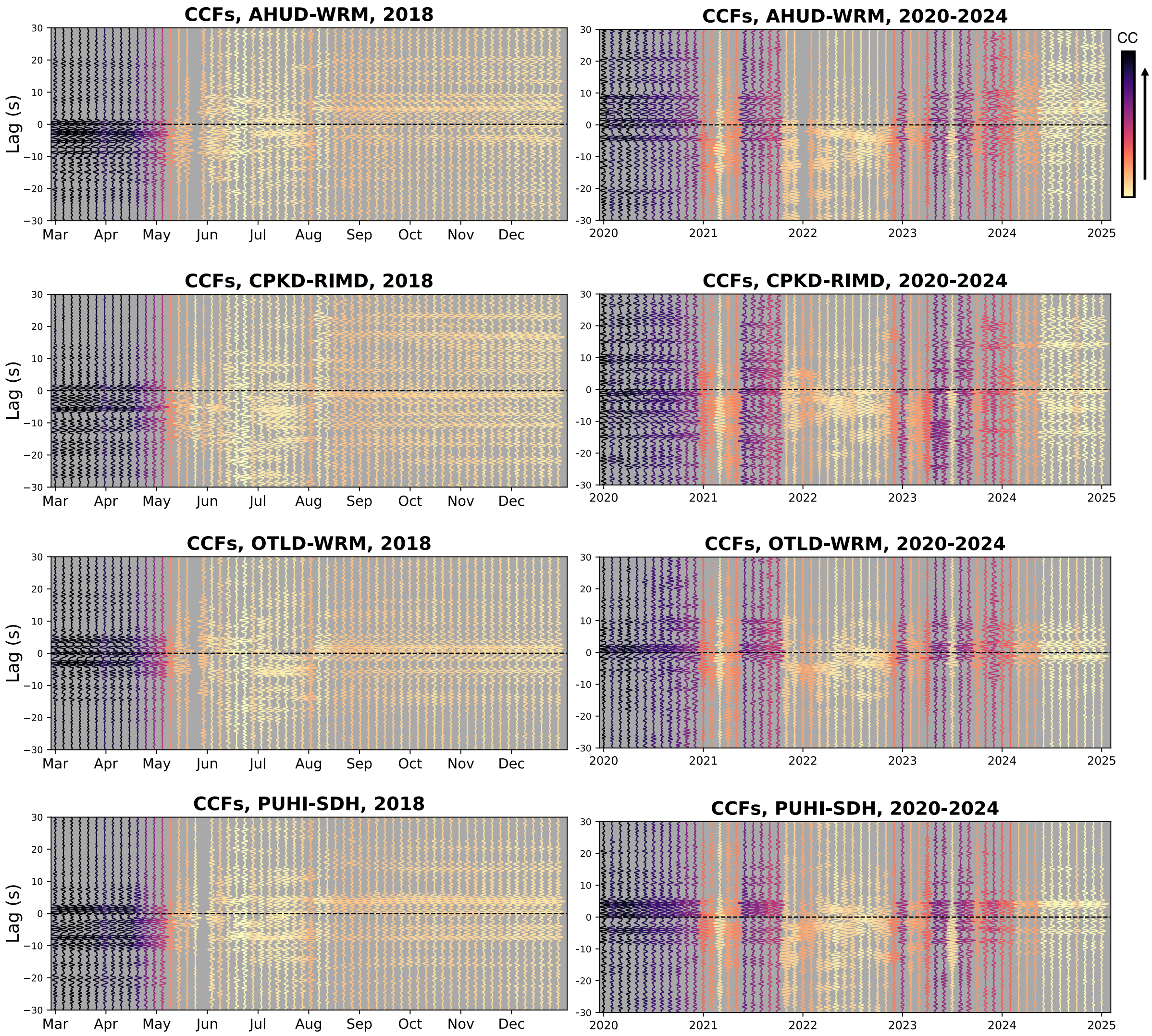

Figure S3: Temporal cross-correlation functions (CCFs) for four additional station pairs for the time periods 2018 and 2020-2024. The CCFs are color-coded according to their correlation coefficients, with darker colors denote higher CC. The reference settings are consistent with those in Figure 4 of the main text.

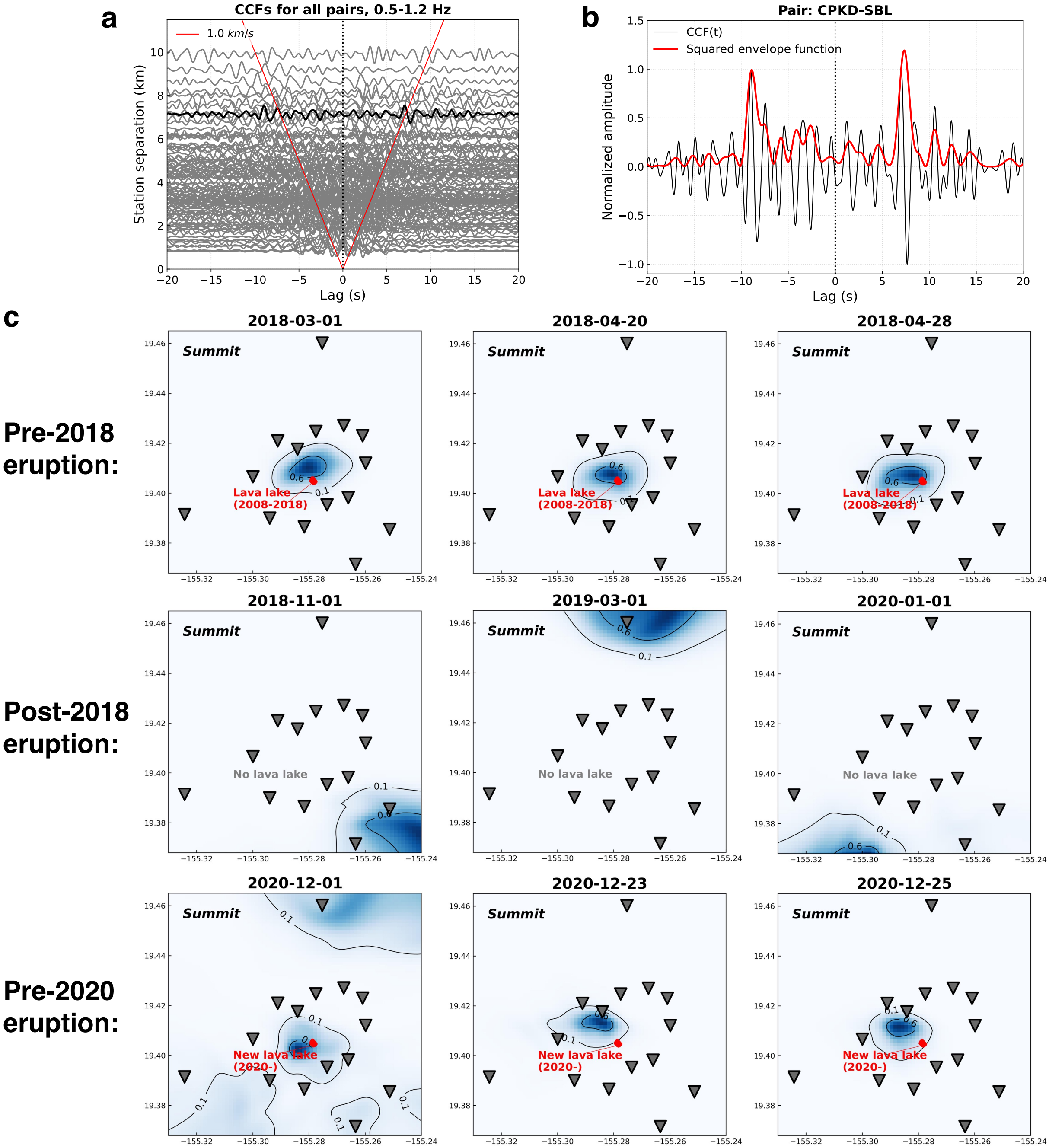

Figure S4: Localization of the ambient noise source energy. (a) Daily (April 1, 2018) example of cross-correlations for all station pairs. Red lines indicate a velocity of 1.0 m/s, marking the arrival of primary wave phases. The black CCF highlights the example pair shown in Panel (b). (b) CCF for pair CPKD-SBL extracted from Panel (a). The red curve shows the squared envelope function used to locate the noise source energy. (c) Spatiotemporal distribution of noise source energy. We present three temporal maps in each row, corresponding to each stage of the summit lava lake. The lava lake location (red dot) is approximated within HC and does not represent real-time lake boundaries.

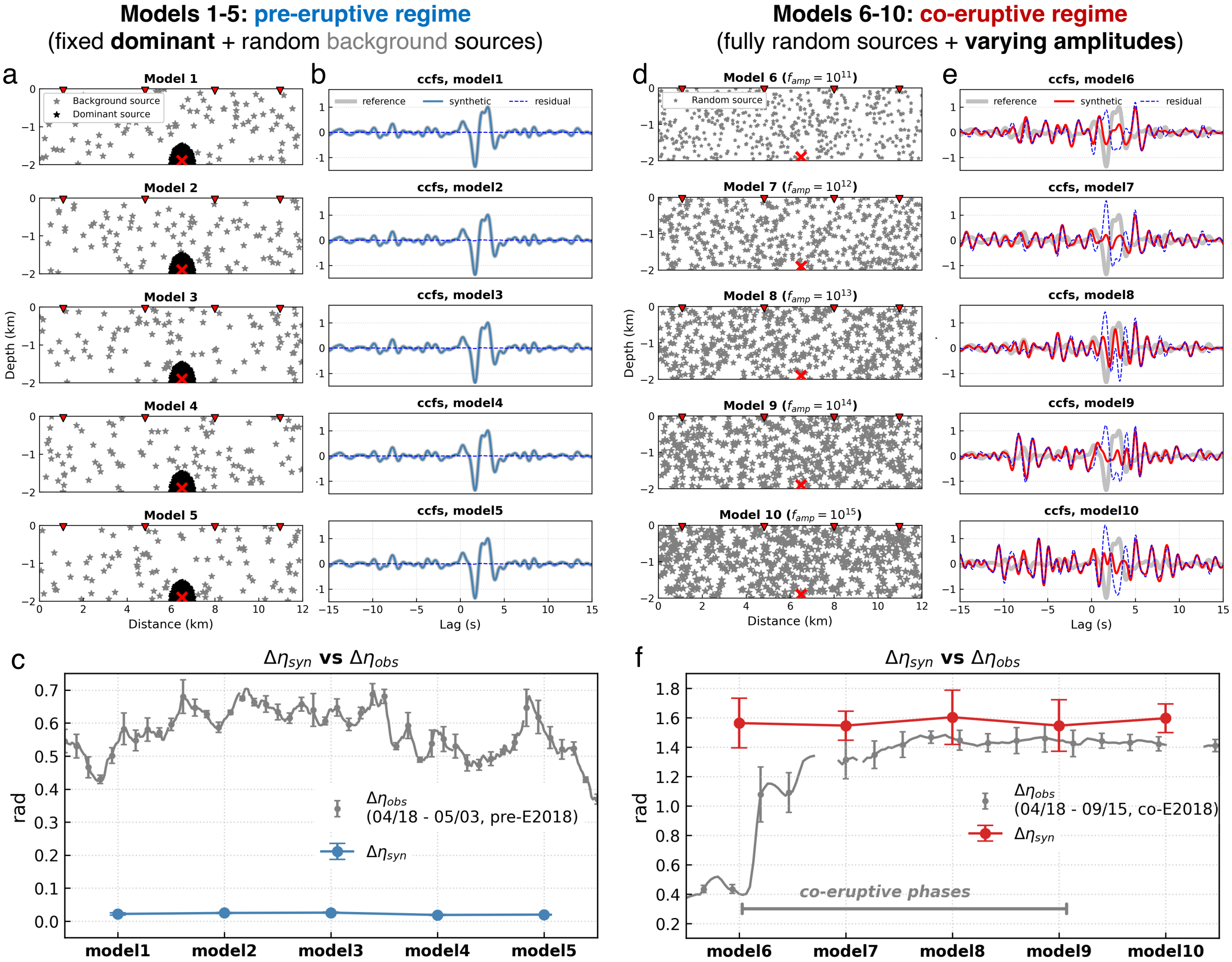

Figure S5: Sensitivity analysis of $\Delta\eta$ to noise-source variability, supporting Table 1 in the main text. (a) Models 1-5 represent moderate perturbations in pre-eruptive regime. (b) Example of cross-correlation functions from the reference model (Supplementary Fig. S13b) and from each test model, together with their residuals. (c) Comparison between synthetic $\Delta\eta$ and pre-eruptive $\Delta\eta$. Each circle represents the mean $\Delta\eta$ with the standard deviation over all four reference-station choices. (d) Models 6-10 represent extreme perturbations in co-eruptive regime (amplitude factors from 10^{11} to 10^{15}). (e)-(f) Same as (b)-(c), but for Models 6-10. Additional details of these tests are provided in "Methods" of the main text.

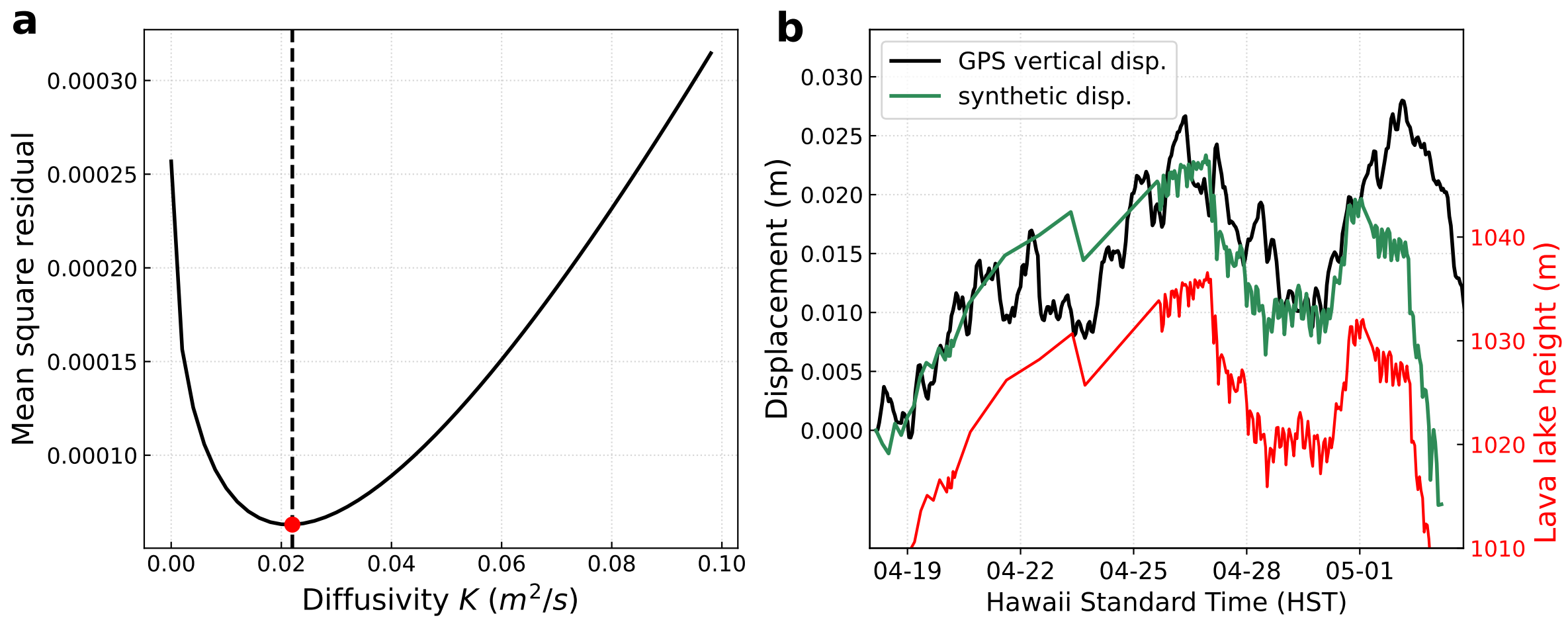

Figure S6: Estimation of hydraulic diffusivity value and simulation of ground displacement. (a) A grid search to determine the value of hydraulic diffusivity (κ in Equation S1). The best-fit value of κ is estimated to be 0.022 m^2/s. (b) The observed GPS vertical displacement at the GNSS station CRIM (in black), and simulated pressure-driven displacement (in green) combining the optimal κ with Equations S1 and S3. The GPS observation is derived from the 5-minute final solutions (HVO, 2008; Blewitt et al., 2018), and we further smooth it using a 3-hour running mean. The lava lake height changes (in red) are used as input pressure perturbations for the simulation.

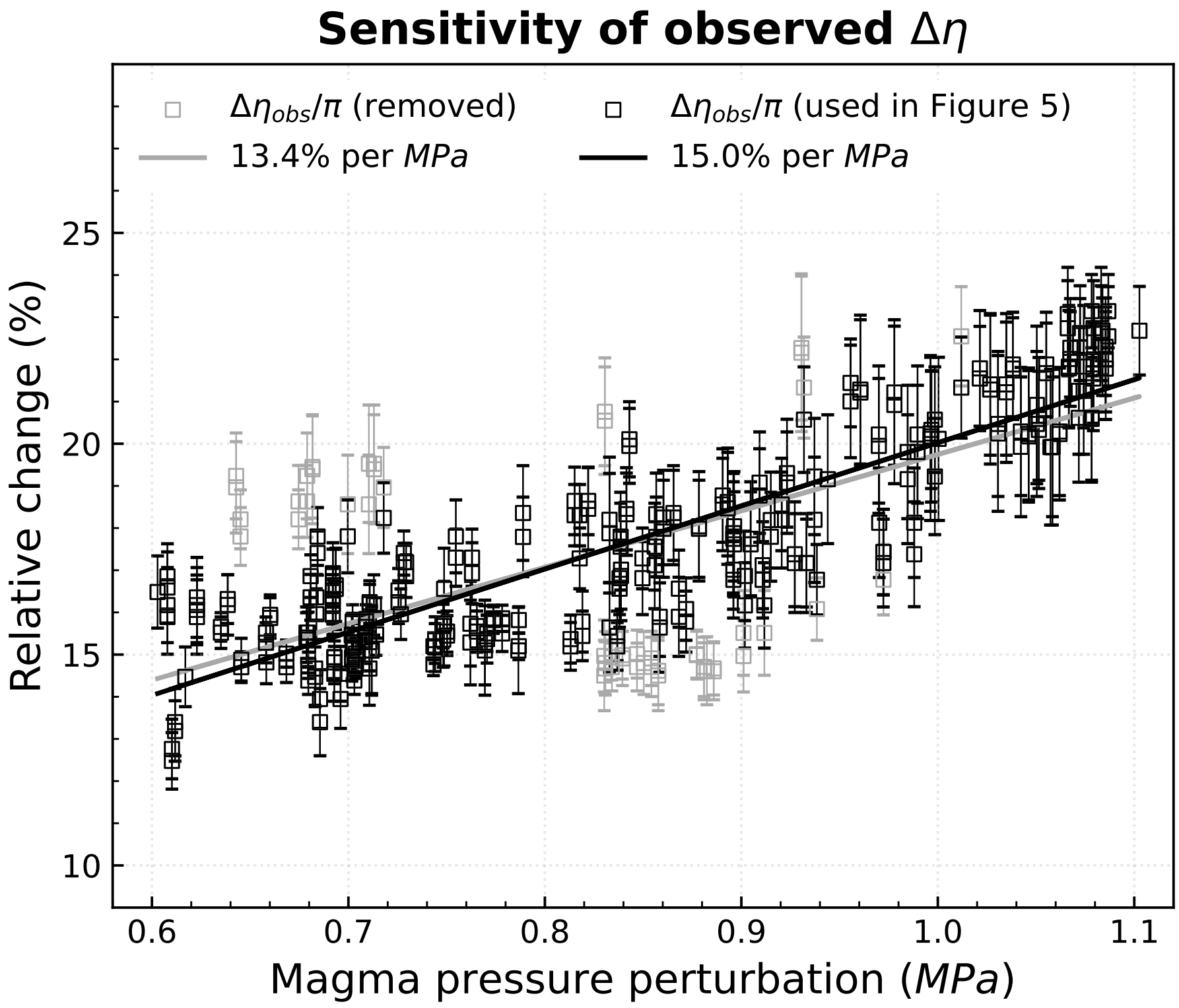

Figure S7: Linear regression of observed geometric phase change ($\Delta\eta$) versus magma pressure perturbation. $\Delta\eta$ samples within the confidence threshold (Z-score $\leq$ 1.5, black squares) are used to fit the slope in Figure 5b of the main text, while outliers (gray squares) are excluded. The fitted slope including the outlier samples (gray line) yields a 13.4% per MPa, which is slightly lower than the current 15.0% (black line). Samples with pressure perturbations lower than 0.6 MPa are additionally excluded due to the sparse sampling of lava lake heights.

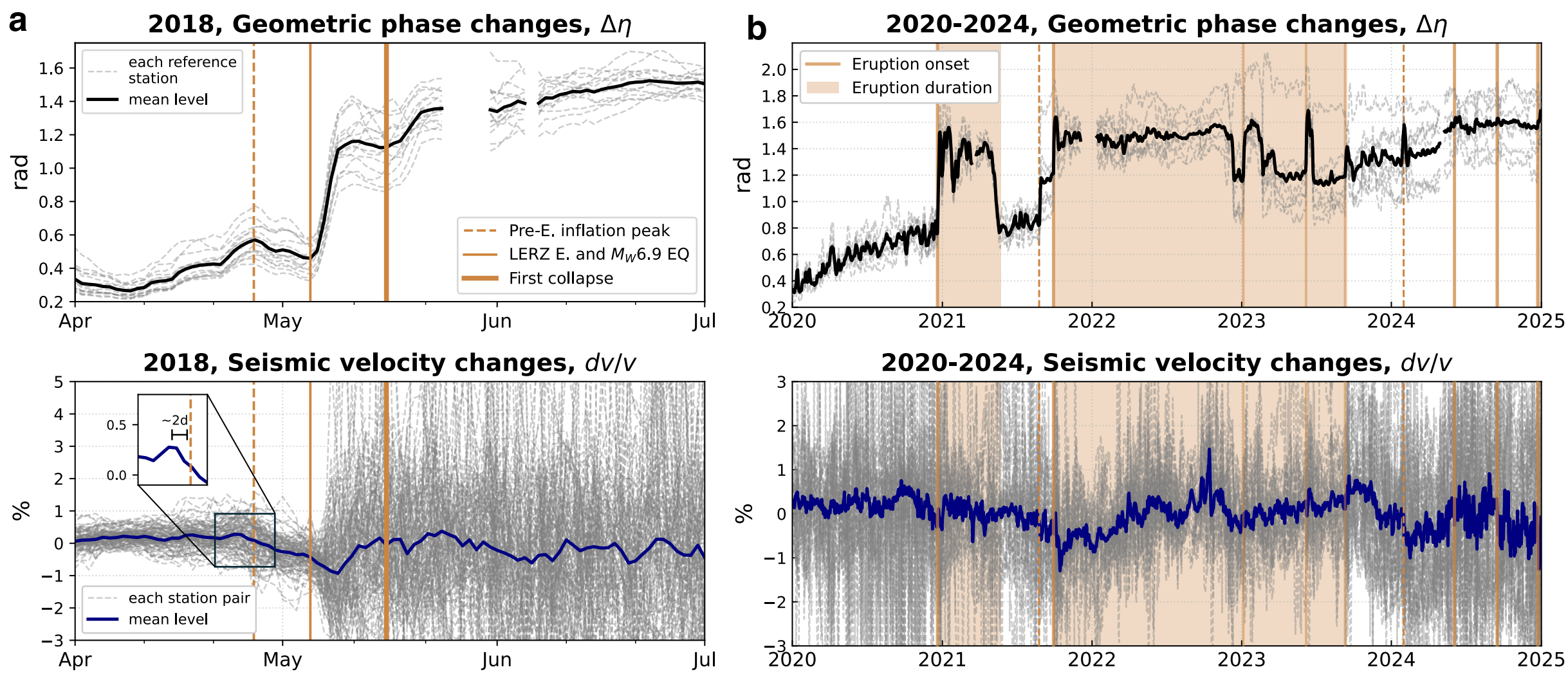

Figure S8: Comparison of observations in $\Delta\eta$ and relative seismic velocity (dv/v). (a) and (b) show the two analysis periods: 2018 and 2020-2024. The geometric phase $\Delta\eta$ and eruption chronology are same as in Figures 2 and 3a of the main text. The measured dv/v are shown for each station pair (gray dashed curves) and their mean level (blue bold curves). Details on dv/v measurements are provided in Supplementary Text S1.

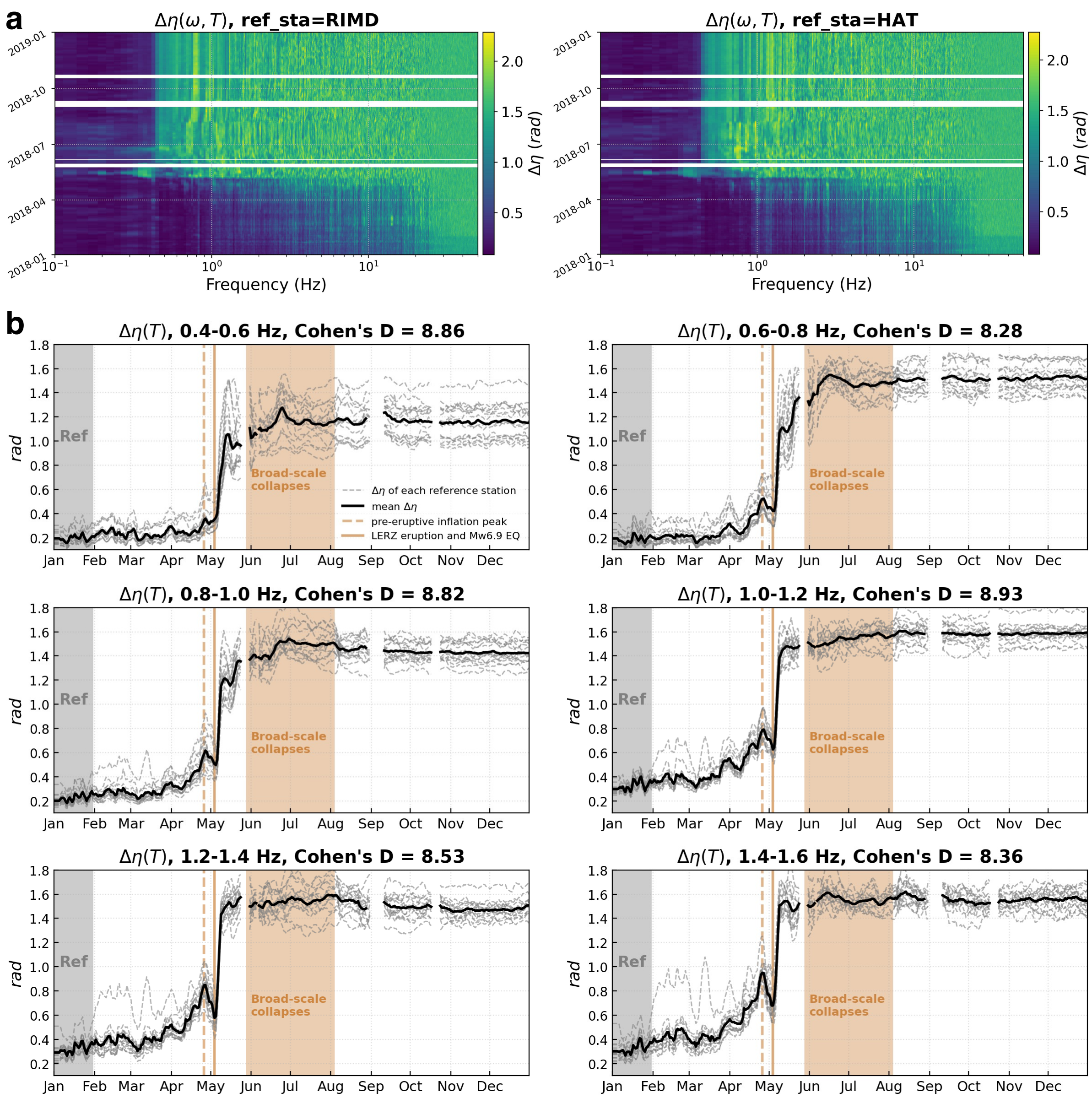

Figure S9: Geometric phase ($\Delta\eta$) measurements in 2018. (a) Two examples of time-frequency functions for $\Delta\eta(\omega,T)$ measured by using reference stations RIMD and HAT. (b) The $\Delta\eta(T)$ time series averaged over six frequency ranges, each with an interval of 0.2 Hz. Other eruptive symbols are same to Figure 2 in the main text.

Cohen's D: Quantifies the separation between eruption (er) and background (bg) signals (split on May 3, 2018), defined as: $D = \frac{\mu_{er}-\mu_{bg}}{\sigma_{pooled}}$ and $\sigma_{pooled} = \sqrt{\frac{(n_{er}-1)\sigma_{er}^2+(n_{bg}-1)\sigma_{bg}^2}{n_{er}+n_{bg}-2}}$. μ and σ are mean and standard deviation of each period, n is the sample size. We separate the background and eruption signals at May 3, 2018, representing the onset of LERZ eruption.

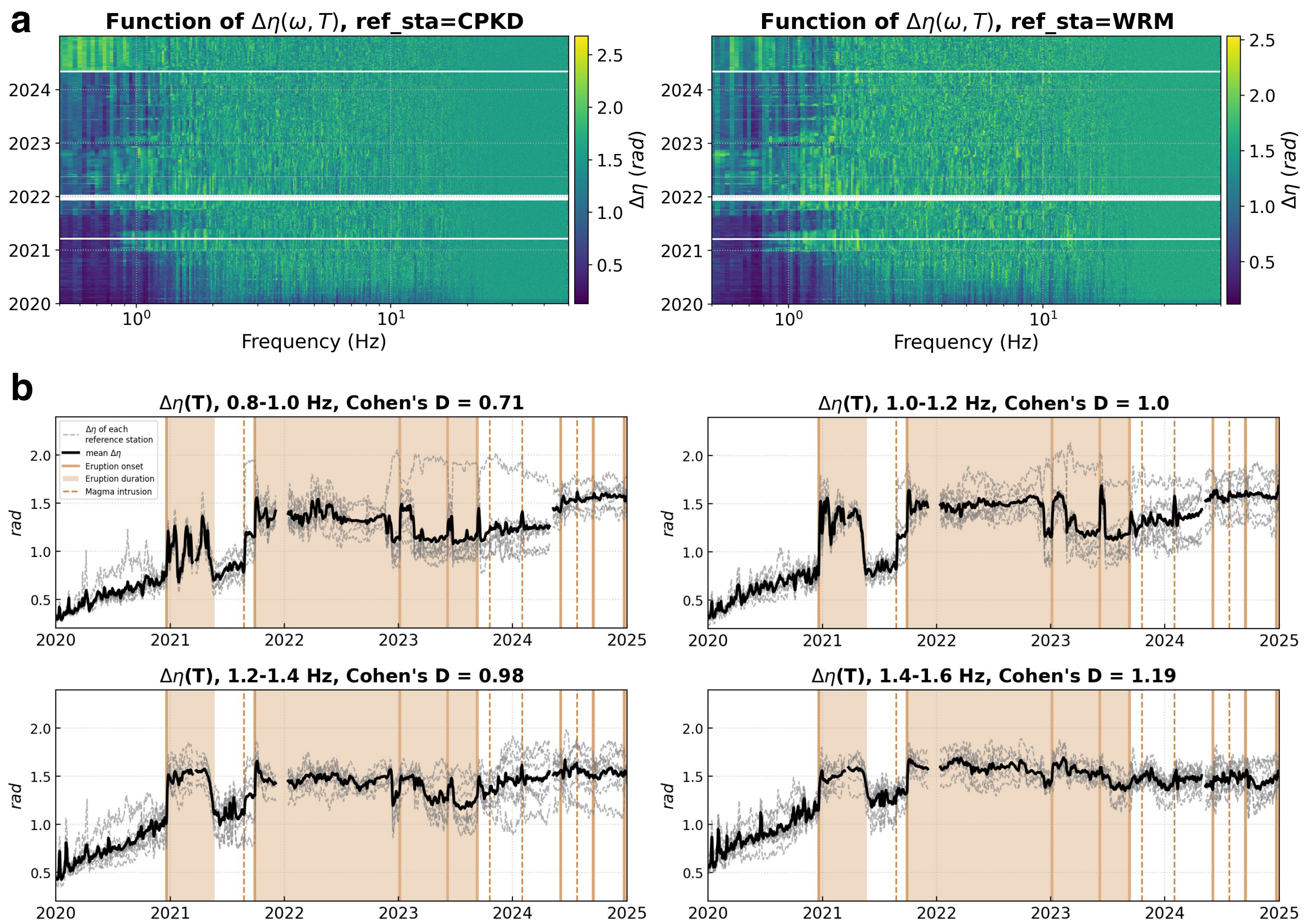

Figure S10: Geometric phase ($\Delta\eta$) measurements from 2020 to 2024. (a) Two examples of time-frequency functions for $\Delta\eta(\omega, T)$ measured by using reference stations CPKD and WRM. (b) The $\Delta\eta(T)$ time series averaged over four frequency ranges, each with an interval of 0.2 Hz. Other eruptive and intrusive symbols are same to Figure 3a in the main text. Cohen's D values are calculated following the same procedure as in Supplementary Fig. S9, except that the eruption signals include all identified eruptive episodes and intrusive activities in 2020-2024.

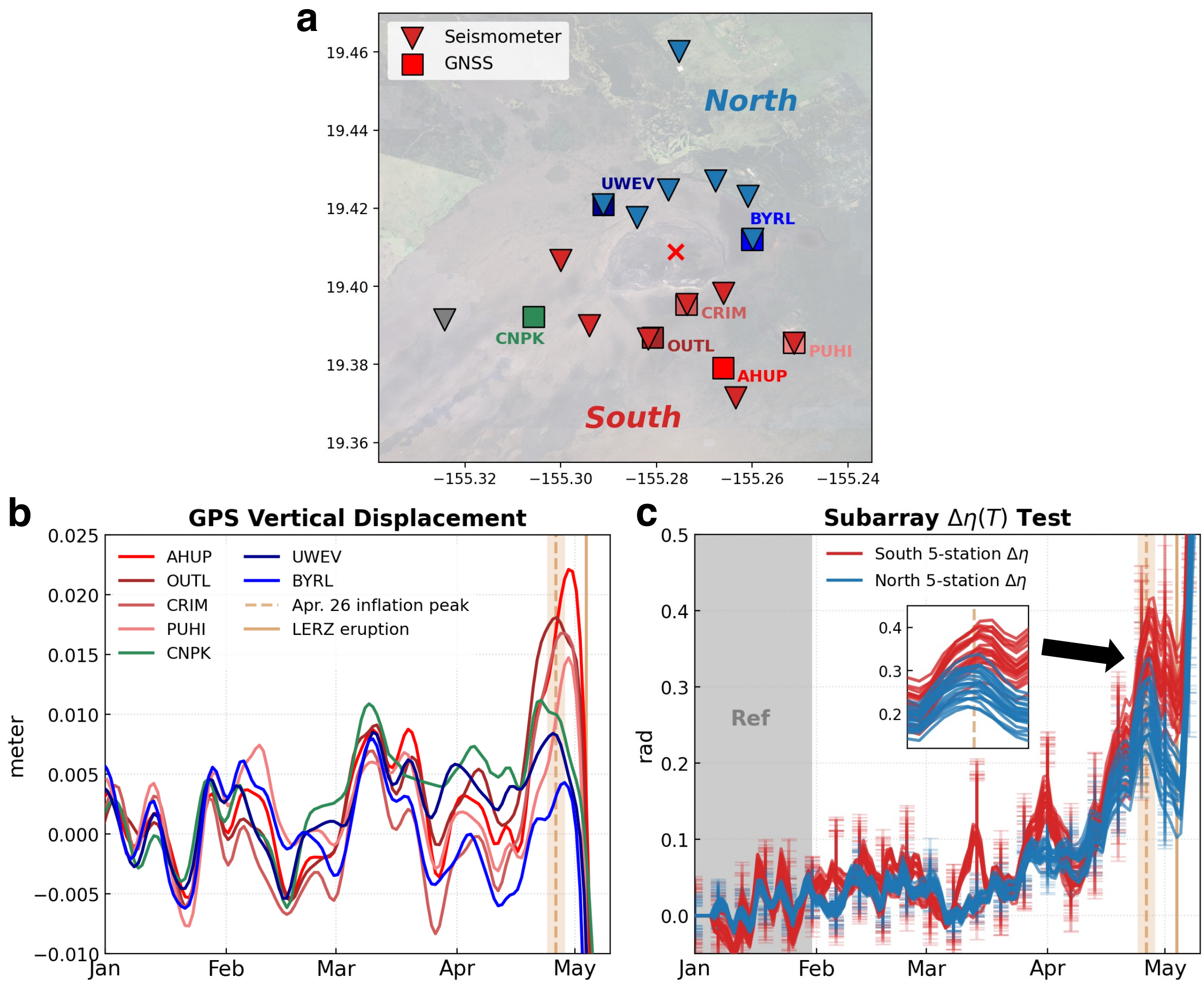

Figure S11: Subarray $\Delta\eta$ test. (a) Distribution of 14 seismic stations and 7 GNSS stations, grouped into southern (red) and northern(blue) groups. GNSS stations are labeled by their codes. (b) Vertical ground displacement time series recorded by 7 GNSS stations, with colors consistent with Panel (a). The time series are smoothed by applying a gaussian filter with the double standard deviation. (c) Subarray-level $\Delta\eta$ time series for all 42 5-station subarrays, shown for the southern (red) and northern (blue) groups. The inset highlights the larger $\Delta\eta$ amplitudes observed in the southern group during the pre-eruptive inflation peak.

Subarray $\Delta\eta$ analysis. We performed a subarray-based $\Delta\eta$ analysis focusing on the pre-eruptive inflation peak on April 26, 2018. 14 seismic stations were selected and grouped into southern and northern groups (red and blue triangles in Panel a). Staion CPKD (gary triangle in Panel a) was excluded due to its remote location. Within each group, all possible 5-station subarrays were constructed, yielding 21 distinct subarrays per group. We also show the distribution of 7 GNSS stations across the summit (squares in Panel a), which provide vertical ground displacement records (Panel b) for comparing the $\Delta\eta$ evolution. $\Delta\eta$ was then calculated independently for each 5-station subarray using identical parameter settings as in the 2018 analysis. In Panel c, each subarray-level $\Delta\eta$ is presented as the mean level, with

the standard deviation, across all 5 reference station settings. This procedure allows evaluation of both the mean behavior and variability of $\Delta\eta$ within southern and northern groups, without modifying the underlying geometric phase formulation.

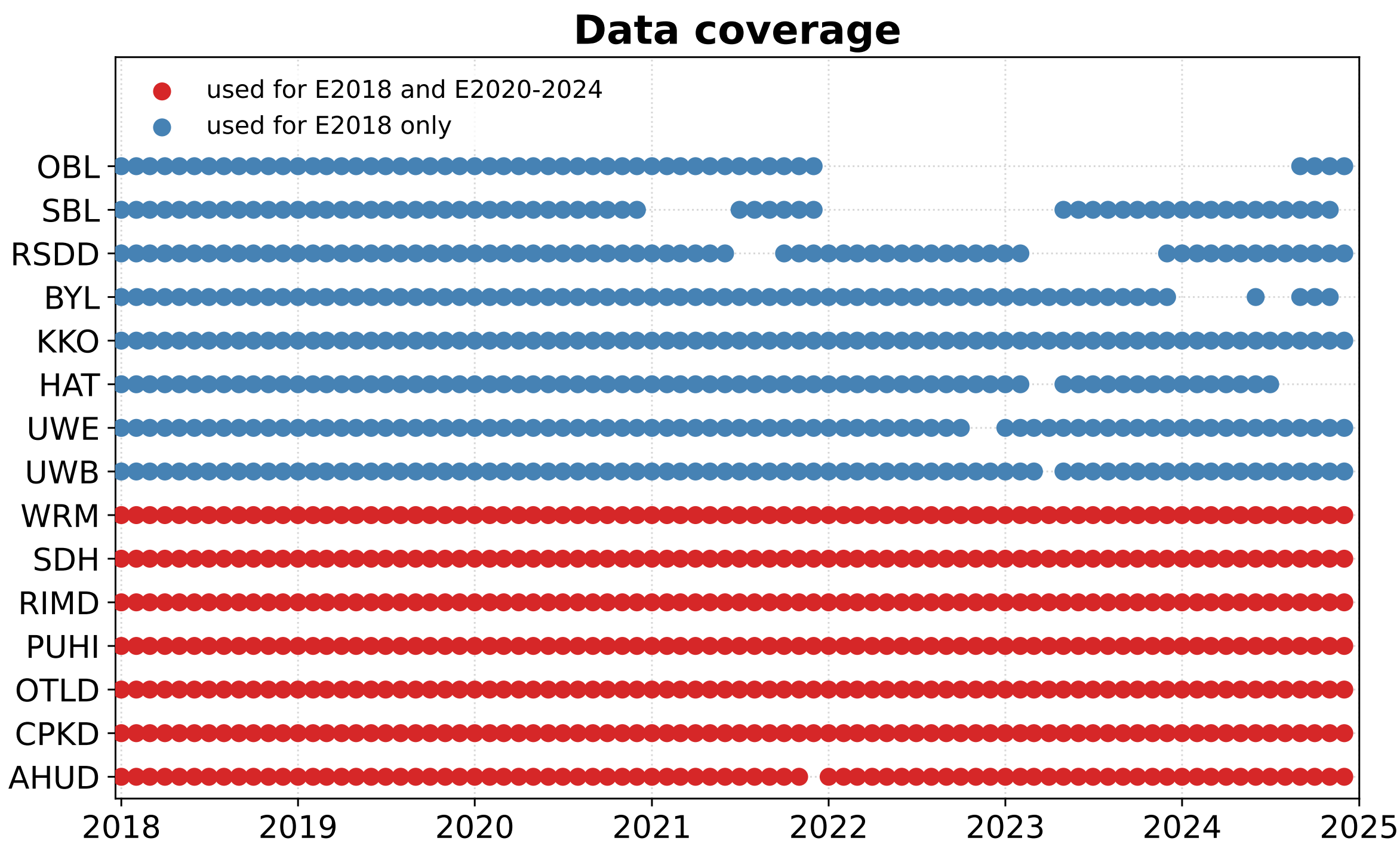

Figure S12: Data coverage for the vertical component seismograms from 15 stations in our study area. All 15 stations were used for the geometric phase analysis in 2018 (Figure 2 in the main text). For the investigation from 2020 to 2024 (Figure 3 in the main text), only 7 stations (red dots) were included, while the others (blue dots) were excluded due to missing data recordings during key eruptive periods. Small recording gaps within one month are not shown.

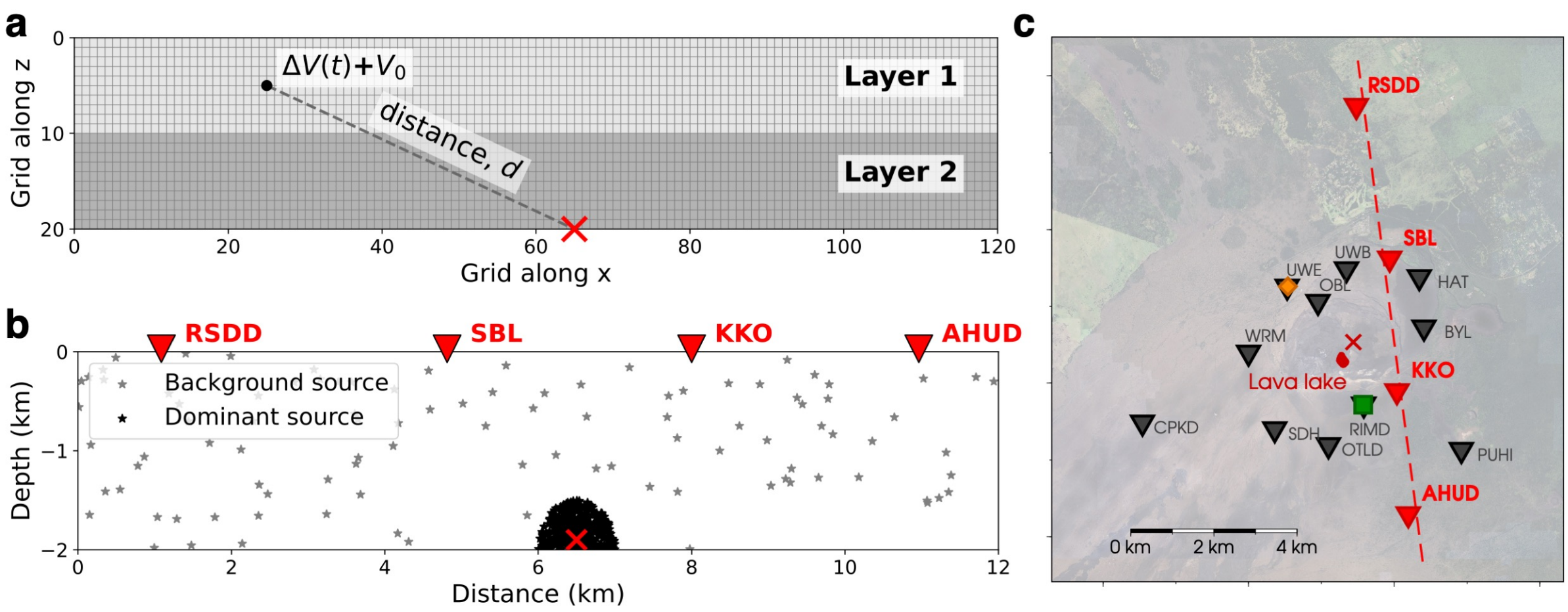

Figure S13: Model construction for the numerical simulation of $\Delta\eta$. (a) Input tomographic file with velocity perturbations. At each grid point, the computed velocity change $\Delta V(t)$ is applied to the initial two-layer 1D velocity profile V_0. $\Delta V(t)$ (both ΔV_p and ΔV_s) is computed from Equation S2, incorporating distance d from the magma reservoir center (red cross). (b) Configuration of 500 point sources in the reference model, including 400 dominant volcanic tremor sources and 100 background sources. (c) The 4 selected stations span a linear geometry in the field.

a

Initial velocity profile

	Depth, *km*	Vp_0, *m/s*	Vs_0, *m/s*
**Layer 1**	0-1	2514	1500
**Layer 2**	1-2	3254	1533

b

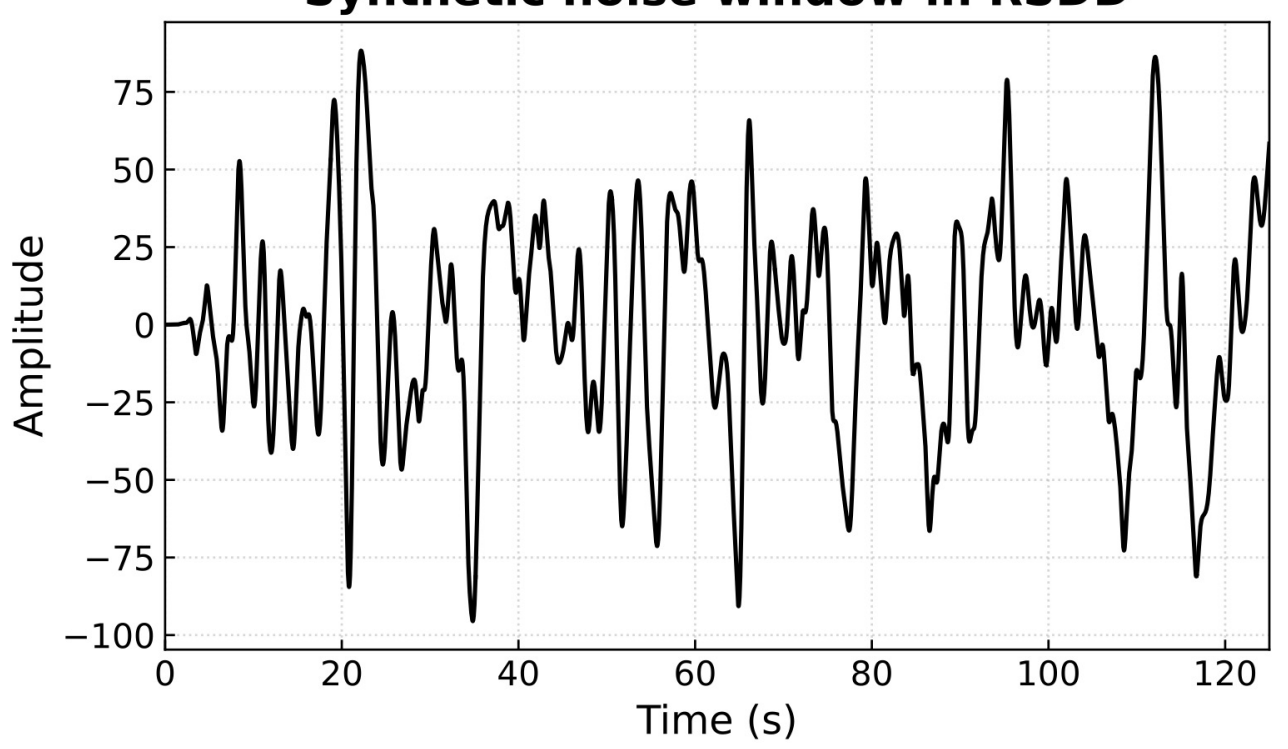

c

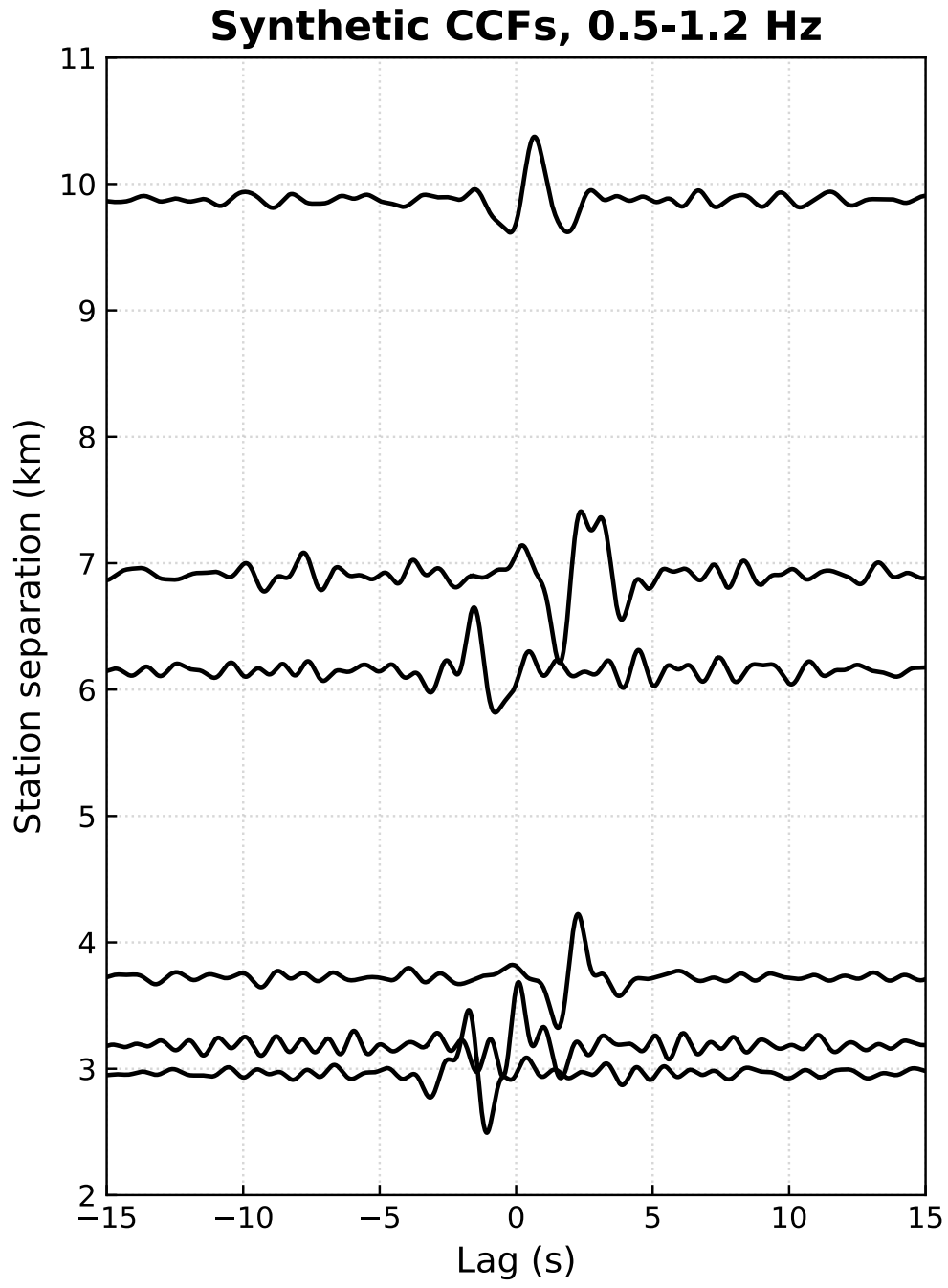

Figure S14: Simulated seismograms. (a) The 1-D initial velocity profile for the study area (Lin et al., 2014) is adopted as the reference model, and is used in the velocity perturbation calculations shown in Supplementary Fig. S13a. (b) A synthetic 125-second noise window from the vertical component of station RSDD. (c) Examples of cross-correlations between all station pairs using synthetic noise. Seismograms in Panels (b) and (c) are from the simulated time step at 2018-04-18, 04:00:00.

References

Anderson, K. R., Johanson, I. A., Patrick, M. R., Gu, M., Segall, P., Poland, M. P., ... Miklius, A. (2019). Magma reservoir failure and the onset of caldera collapse at kīlauea volcano in 2018. *Science*, *366*(6470), eaaz1822. doi: 10.1126/science.aaz1822

Blewitt, G., Hammond, W. C., & Kreemer, C. (2018). *Harnessing the GPS data explosion for interdisciplinary science.* ([Dataset]) doi: https://doi.org/10.1029/2018EO104623

Clarke, D., Zaccarelli, L., Shapiro, N. M., & Brenguier, F. (2011, 08). Assessment of resolution and accuracy of the Moving Window Cross Spectral technique for monitoring crustal temporal variations using ambient seismic noise. *Geophysical Journal International*, *186*(2), 867-882. doi: 10.1111/j.1365-246X.2011.05074.x

HVO, U. (2008). *Hawaii GPS Network - CRIM-Crater Rim P.S.* The NSF GAGE Facility operated by EarthScope Consortium. (Accessed on May 2025, [Dataset]) doi: https://doi.org/10.7283/T5RR1WGN

Lecocq, T., Caudron, C., & Brenguier, F. (2014). MSNoise, a python package for monitoring seismic velocity changes using ambient seismic noise. *Seismological Research Letters*, *85*(3), 715-726. ([Software]) doi: 10.1785/0220130073

Lin, G., Shearer, P. M., Matoza, R. S., Okubo, P. G., & Amelung, F. (2014). Three-dimensional seismic velocity structure of mauna loa and kilauea volcanoes in hawaii from local seismic tomography. *Journal of Geophysical Research: Solid Earth*, *119*(5), 4377-4392. doi: https://doi.org/10.1002/2013JB010820

Murnaghan, F. D. (1937). Finite deformations of an elastic solid. *American Journal of Mathematics*, *59*(2), 235–260. Retrieved 2025-06-03, from `http://www.jstor.org/stable/2371405`

Patrick, M., Orr, T., Swanson, D. A., Houghton, B. F., Wooten, K., Desmither, L., ... Fee, D. (2021). Kīlauea's 2008–2018 summit lava lake—chronology and eruption insights. *US Geological Survey professional paper*, *1867*(A). Retrieved from `https://par.nsf.gov/biblio/10240311`

Patrick, M., Younger, E., & Tollett, W. (2019). Lava level and crater geometry data during the 2018 lava lake draining at kīlauea volcano, hawaii. *US Geol. Surv. Data Release*. ([Dataset]) doi: 10.5066/P9MJY24N

Tsai, V. C. (2011). A model for seasonal changes in gps positions and seismic wave speeds due to thermoelastic and hydrologic variations. *Journal of Geophysical Research: Solid Earth*, *116*(B4). doi: https://doi.org/10.1029/2010JB008156
