# Supplementary material for: Geometric phase sensing using seismic waves for comprehensive volcano monitoring at Kı̄lauea Hawaii
Source: Nat Commun. 2026 Jun 26;17:7988. doi: 10.1038/s41467-026-73998-x (PMC13448816; doi:10.1038/s41467-026-73998-x)
Supplement: Supplementary file 2 [file 41467_2026_73998_MOESM2_ESM.pdf]

nature portfolio

Peer Review File

Geometric phase sensing using seismic waves for comprehensive volcano monitoring at Kīlauea Hawaii

Corresponding Author: Dr Bingxu Luo

This file contains all reviewer reports in order by version, followed by all author rebuttals in order by version.

Version 0:

Reviewer comments:

Reviewer #1

(Remarks to the Author)
The paper presents a new approach—geometric phase sensing (Δη)—derived from topological acoustics, applied to ambient seismic noise at Kīlauea volcano. The authors claim that Δη captures magmatic pressurization, caldera collapse, and recovery phases across multiple eruptions (2018–2024), outperforming traditional dv/v methods in robustness and sensitivity. Numerical simulations suggest Δη responds to subsurface pressure at ~15% per MPa.

The manuscript is clearly written and engaging. The geometric-phase framework is conceptually novel and may indeed encapsulate multi-station coherence changes efficiently. However, I remain unconvinced regarding its physical interpretability and independence from source effects.

Having that said, if the authors can convincingly demonstrate that Δη is not merely a proxy for broadband coherence loss this would be an interesting publication in Nature Communications.

Best regards,
Anne Obermann

Below, I outline my main reservations.

Major Points
1. Attribution of Δη Changes: Subsurface vs. Noise-Source Effects
While the authors assert that Δη "remains detectable despite unstable noise fields," the evidence presented does not convincingly distinguish subsurface medium changes from variations in the spatial or temporal distribution of ambient noise sources.
• Temporal cross-correlation functions (Fig. 4a,d) show clear coherence loss during eruptive phases, likely due to the Mw 6.9 earthquake and elevated seismicity, suggesting that at least part of the Δη signal could reflect noise-field instability.
• Noise-field localization (Fig. 4c,f) shows significant migration of energy sources before and after eruptions (from summit to distal sites and back). These shifts coincide with the largest Δη variations, yet no quantitative test demonstrates that Δη is unaffected by such source evolution.
• Matched Field Processing maps confirm evolving tremor sources, hence please show that comparable Δη fluctuations would not arise from source movement alone.
• The numerical simulations assume a fixed source distribution, thus testing only sensitivity to medium changes—not to realistic source variability.
Recommendation:
Provide a quantitative sensitivity analysis where source geometry and intensity is perturbed under constant medium properties to assess Δη variability due solely to source changes.

2. Demonstrating that Δη is not a proxy for Coherence Loss
Figures 4b,e reveal that Δη extremely closely tracks CCF coherence. The manuscript needs to show what additional information Δη provides beyond a conventional coherence or waveform-similarity analysis.
• The geometric phase depends on path evolution, similar to waveform similarity, already used in seismic interferometry and non-destructive testing.

• By averaging over station pairs, Δη sacrifices spatial information that could help disentangle source from medium effects.
• Changes in waveform similarity (and given the correlation also Δη) can reflect either structural perturbations (altered scattering, fracturing) or source variability; this dual sensitivity must be explicitly addressed (see point 1).
Recommendation:
Quantitatively compare Δη to broadband coherence loss and waveform similarity metrics. Clarify whether Δη captures independent physical information or is a repackaged coherence measure.

3. Physical interpretability and mechanistic link
The conceptual step from "geometric phase rotation in Hilbert space" to "magma-induced medium stiffening" remains insufficiently justified.
• The poroelastic model (Eq. 6) is used post hoc to fit observations, but it does not explain how the geometric phase—constructed from complex CCF vectors—relates physically to volumetric strain or pressure-induced velocity changes.
• The conversion from lava lake height to pressure ($\Delta P_m \approx \rho g \Delta h$) and then to Δη is empirical, lacking a derivation that links ΔV/V perturbations to measured phase rotations.
Recommendation:
Clarify the model connecting seismic velocity perturbations to geometric phase changes. Consider testing subsets of stations to evaluate how spatial averaging affects sensitivity and interpretability.

Additional Comments
• Statistical validation: No quantitative test supports the significance of detected changes. Apply a change-point or time-series analysis (e.g., Sánchez-Pastor 2018) to confirm statistically meaningful shifts.
• Comparison to dv/v and waveform similarity methods: The current comparison is limited. The Moving-Window Cross-Spectral (MWCS) technique assumes homogeneous changes and is not ideal here. A frequency–time analysis contrasting reference-biased and unbiased approaches would be more informative (see Obermann and Hillers 2019).
• Figures 2–3: Include noise-power or SNR plots alongside Δη to show whether phase variations coincide with noise-intensity fluctuations.
• Short-term analyses: The use of 1-hour CCF stacking requires statistical justification, especially during periods of low coherence.

References

Sanchez-Pastor et al. (2018) Detecting and locating precursory signals during the 2011 El Hierro, Canary Islands, submarine eruption, GRL
Obermann and Hillers (2019) Seismic Time-Lapse Interferometry across Scales, Advances in Geophysics, Vol 60, doi: 10.1016/bs.agph.2019.06.001

(Remarks on code availability)
I have downloaded the example data set (CCFS) and could run the the python code for the geometric phase computation. Readme files and instructions were given.

I have not run the other codes (MFP, Correlation, Simulation) but they also seem well documented.

Reviewer #2

(Remarks to the Author)
Review of "Geometric phase sensing using seismic waves: A new tool for comprehensive volcano monitoring at Kilauea, Hawai'i" by Luo et al.

Over the past 10-20 years cross-correlating ambient seismic records for time-lapse monitoring has emerged as a major subdiscipline in seismology. I think a challenge for this field has been that there are so many causes for the types of changes being measured as relative velocity changes, called dv/v. In that way it reminds me of older studies of seismic b-values: so many things can cause variations in it, essentially all subsurface processes one can think of. Studies have shown that relative velocity changes or dv/v can be caused by hydrologic variations, strong ground shaking due to large earthquakes, magma intrusion or extrusion, seasonal or environmental effects, and so on; unraveling which mechanism is the underlying cause for a particular measurement can be difficult. In this paper, the authors introduce a new type of processing scheme called geometric phase sensing and apply it to detecting time-lapse changes in ambient seismic records at Kilauea volcano during its recent eruptions. The method is original, interesting, and a potentially paradigm-shifting way of processing ambient noise correlations. However, after reading the paper, I came away with questions and comments that I list in the following. I give the main ones first and then minor ones afterwards.

-My first comment is about the terminology of "geometric phase." This term has already been used in seismology and I'm unclear whether it's being used in the same sense in this paper. For example, the term "geometric phase" is synonymous with "Berry phase" (after the physicist Michael Berry) and has wide applicability in many branches of physics, both quantum and classical. Here is the wikipedia page about it:
https://en.wikipedia.org/wiki/Geometric_phase
In classical physics, a common example of the geometric phase is the motion of a Foucault pendulum due to the Earth's

rotation. In seismology, the term “geometric phase” or “Berry phase” has similarly been used to describe changes in seismic waves, in particular the polarization of seismic shear waves, due to the rotation of the Earth or anisotropy. These are a few seismic papers on this phenomenon:
Tromp, J. & Dahlen, F. A., 1992. The Berry phase of a slowly varying waveguide, Proc. R. Soc. Lond., A, 437, 329–342.
Tromp, J. (1994), Surface-Wave Propagation On A Rotating, Anisotropic Earth. Geophysical Journal International, 117: 141-152.
Snieder, R., C. Sens-Schönfelder, E. Ruigrok, and K. Shiomi (2016), Seismic shear waves as Foucault pendulum, Geophys. Res. Lett., 43, 2576–2581.
When I agreed to review this paper, having only seen the title, I expected something like what I knew as the “geometric phase” in this sense and I’m unsure if the type of analysis in this paper is similar. If it isn’t, and my gut feeling is that it is not, then I think there needs to be a statement near the beginning of the paper that clarifies this use of the term “geometric phase” is different from these previous uses. However, given that the term “geometric phase” is already used in seismology, I would recommend changing the terminology if all possible to avoid confusion. An alternative I can think of is that instead of “geometric phase sensing” one could say “topological acoustic sensing.” If it is the case that the term “geometric phase” is being used in this paper in a way that this consistent with prior uses of it in seismology, then it’s ok to keep using it but again I think there needs to be an explanation early on about how this is connected things like S-wave polarization anomalies due to the Earth’s rotation.

-There is a key reference missing in this paper, which is this one:
Muzellec, T., Lesage, P., Caudron, C., & Got, J.-L. (2023). Migration of mechanical perturbations estimated by seismic coda wave interferometry during the 2018 pre-eruptive period at Kīlauea volcano, Hawaii. Journal of Geophysical Research: Solid Earth, 128, e2022JB026224.
I bring it up because in Figure 5 of the paper by Muzellec et al. they show the peak of dv/v occurring on about April 26, 2018 (labeled t1 in their figure) similar to the peak in \delta\eta (geometric phase change) shown in this paper in Fig. 2. It’s important because the authors argue in the Discussion section at lines 249-251 that the observation of this peak in \delta\eta is an indication of its advantage over dv/v:
“For instance, the April 26, 2018 ground inflation peak (Day -4 in Figure 2) was not captured by previous dv/v [42] or our reproduction (2-day lag, inset of Supplementary Fig. S7a), likely due to complex pressure accumulation and reduced bulk edifice strength.”
In contradiction to this statement, the peak has been captured in a previous dv/v study by Muzellec et al. and they show its exact occurrence in time varies depending on the distance east from the caldera, a precise detail which the authors do not discuss in their paper.

-My overall summary of the geometric phase method is that it is an interesting and fresh approach to time-lapse ambient seismic studies. But there are challenges and tradeoffs. One I can see is that the geometric phase method is very general and one always gets a measurement (a positive advantage), in contrast to dv/v which requires some level of coherence and stability of the noise source. However, there is a sensitivity kernel for dv/v that relates it spatially to physical properties in the subsurface - see the cited paper by Obermann et al. (2013). Obermann et al. also show another independent type of measurement called decorrelation that has a sensitivity kernel related to the changing scattering properties of the subsurface in contrast to changing velocity as for the dv/v. The geometric phase has increased sensitivity compared to dv/v (and presumably decorrelation as well) as the authors show in Fig. 5b, but the tradeoff is that all these different physical changes, like changing velocity or scattering or even changing noise source, are accumulated into \delta\eta. So it will be very difficult, if not impossible, to find meaningful sensitivity kernels for \delat\eta and to spatially invert for where the changes are happening in the subsurface as can be done for dv/v and decorrelation. So the tradeoff is increased sensitivity to change at the expense of reduced ability to spatially map where the change is occurring. It’s also interesting to see the similarity between \delat\eta and the simple correlation coefficient (actually its inverse) as the authors show in their Fig. 4. This shows that \delat\eta is really a fairly simple measurement similar to the simple correlation coefficient (note that this simple correlation coefficient is different from decorrelation as discussed by Obermann et al.). Regarding the need for coherence and noise source stability for dv/v measurements, I’m reminded in a way of similar challenges in InSAR processing in satellite remote sensing. In InSAR, there is a co-registration issue where measurements can’t be made if coherence between the two satellite images is lost. This is a similar issue that can afflict dv/v changes and make them impossible to measure. What it makes me wonder is if there may be applications of the geometric sensing method to InSAR processing as well in the future. So as I said earlier there are tradeoffs that should be acknowledged but the geometric sensing method is a fresh approach and opens up new avenues for further development in the future.

-The authors point out that there is a discrepancy of 0.3 in their Figure 5 between observed and synthetic \delta\eta, but what are possible explanations for that?

-The authors consider several different frequency bands in different parts of the paper like 0.6-1 Hz, 0.5-1.2 Hz, and 1-1.2 Hz. In the supplementary info they show that the precise selection of these bands is not overly critical, which is important since it would be difficult to know the optimal band ahead of time. Have the authors also looked in lower frequency bands that are dominated by classic oceanic microseism noise, like 0.25 Hz and below?

Minor comments:
Line 158: the term Koa's should instead be Koa'e
Line 345: change the word axe to axes
Fig 3 caption: The term “geometric phase” is misspelled. What is meant by the term "future eruptions"? I was confused about what that meant.

(Remarks on code availability)
I did not install or run the codes, I simply reviewed the paper. But I loaded the github webpage and from what I can see everything looks to be in order for someone to use the code reproduce results from the paper.

Version 1:

Reviewer comments:

Reviewer #1

(Remarks to the Author)
I thank the authors for their thorough and constructive revision of the manuscript. They have made substantial efforts to address the major concerns raised in the initial review, and the manuscript has improved significantly in rigor and clarity. Importantly, the limitations of the approach are now presented in a transparent manner.

In my view, the revised version meets the standard for publication. Some interpretational limitations remain, and a fundamental proof of the approach is still lacking; however, these points are now appropriately acknowledged by the authors.

Below, I provide a justification for this assessment based structured along my previously raised major concerns and then a short list of minor revisions that could still be done.

1. Source versus medium effects
The authors have significantly strengthened the manuscript by introducing dedicated source-sensitivity tests, including systematic perturbations of source geometry and amplitude under fixed medium conditions. These new analyses provide valuable quantitative constraints.
In particular, the result that moderate source variability produces Δη amplitudes an order of magnitude smaller than observed pre-eruptive signals is an important and convincing addition. The demonstration that extreme source randomization can reproduce large amplitudes but fails to capture the structured temporal evolution is also informative.
At the same time, the revised manuscript appropriately acknowledges that co-eruptive signals likely reflect coupled source and medium effects, and that a strict decomposition would require additional inversion or source-imaging approaches. I agree with this more cautious interpretation. While the attribution remains non-unique, in my view the current treatment is sufficiently rigorous and transparent for publication.

2. Distinction between Δη and coherence-based metrics
The manuscript now provides a much clearer conceptual and quantitative distinction between Δη and conventional coherence measures. The addition of spike-noise tests, along with quantitative metrics (Cohen's D, RMS, NRMS), is particularly effective in demonstrating the robustness of Δη under waveform corruption.
I find the revised interpretation - that Δη is a higher-order, global observable that is complementary to, but not independent from, coherence - appropriate and convincing.

3. Physical interpretation
The authors have substantially improved the discussion of physical meaning. Importantly, the revised manuscript now clearly states that Δη is not a direct proxy for pressure, strain, or velocity changes, and that the poroelastic simulations are intended as consistency tests rather than predictive forward models.
This clarification resolves a key concern from the original submission. While a formal theoretical framework linking Δη to physical parameters remains lacking, the current level of interpretation seems appropriately cautious and acceptable to me.

4. Spatial interpretability
The addition of subarray-level analyses is a useful step toward exploring spatial sensitivity. The observed differences between station subsets provide encouraging indications that Δη may contain spatial information.
However, the manuscript correctly acknowledges that the current framework does not provide sensitivity kernels or support formal spatial inversion. This limitation should remain clearly stated.

Minor remaining revisions that I want to suggest:
The following minor suggestions are primarily to ensure that the cautious interpretations are consistently reflected throughout the manuscript.
• In the subarray analysis section, it should be clearly state that results are indicative only and not equivalent to spatial inversion or localization.
• In the main text and not only the discussion could be stated that co-eruptive Δη signals remain non-unique and reflect coupled effects
• One explicit sentence could be added acknowledging that Δη is empirically correlated with coherence, even if conceptually distinct.
• the word "Instead" should be removed from line 75. Or the sentence rephrased.

(Remarks on code availability)
I have not reviewed the code, only that it is accessible following the DOI provided.

Reviewer #2

(Remarks to the Author)
I have read the authors' responses to my review comments and have come away satisfied that my questions have been addressed properly. I am happy to recommend the manuscript for publication. My only remaining question, based on scientific curiosity, is whether there could be any other useful measurable quantities besides \Delta\eta as defined in equation (2) of the appendix based on this new methodology.

(Remarks on code availability)

Open Access This Peer Review File is licensed under a Creative Commons Attribution 4.0 International License, which permits use, sharing, adaptation, distribution and reproduction in any medium or format, as long as you give appropriate credit to the original author(s) and the source, provide a link to the Creative Commons license, and indicate if changes were made.
In cases where reviewers are anonymous, credit should be given to 'Anonymous Referee' and the source.
The images or other third party material in this Peer Review File are included in the article's Creative Commons license, unless indicated otherwise in a credit line to the material. If material is not included in the article's Creative Commons license and your intended use is not permitted by statutory regulation or exceeds the permitted use, you will need to obtain permission directly from the copyright holder.
To view a copy of this license, visit https://creativecommons.org/licenses/by/4.0/

We thank the reviewers for their careful and constructive evaluation of our manuscript NCOMMS-25-73884, entitled *"Geometric phase sensing using seismic waves: A new tool for comprehensive volcano monitoring at Kīlauea, Hawai'i"*. These insightful comments have significantly helped us improve the clarity, robustness, and presentation of the work. We have revised the manuscript accordingly and provide a detailed, point-by-point response below. Reviewer comments are reproduced in blue italics, and our responses are provided in black. All figure numbers refer to the revised manuscript unless otherwise specified. Bibliographic references can be found in the manuscript. We sincerely appreciate the reviewers' time and effort, and we hope that our revisions have satisfactorily addressed all concerns.

Response to Reviewer 1:

General Comment: *The paper presents a new approach—geometric phase sensing ($\Delta\eta$)—derived from topological acoustics, applied to ambient seismic noise at Kīlauea volcano. The authors claim that $\Delta\eta$ captures magmatic pressurization, caldera collapse, and recovery phases across multiple eruptions (2018–2024), outperforming traditional dv/v methods in robustness and sensitivity. Numerical simulations suggest $\Delta\eta$ responds to subsurface pressure at 15% per MPa.*

The manuscript is clearly written and engaging. The geometric-phase framework is conceptually novel and may indeed encapsulate multi-station coherence changes efficiently. However, I remain unconvinced regarding its physical interpretability and independence from source effects.

Having that said, if the authors can convincingly demonstrate that $\Delta\eta$ is not merely a proxy for broadband coherence loss this would be an interesting publication in Nature Communications.

Response: We thank the reviewer for the careful evaluation of our manuscript and for recognizing the conceptual novelty of the geometric phase approach. We appreciate the major concerns regarding the influence of source effects and physical interpretability. In the revised manuscript, we have substantially clarified these aspects. Specifically, we (1) expand the theoretical discussion distinguishing $\Delta\eta$ from conventional waveform coherence metrics, (2) demonstrate its robustness under varying ambient noise conditions, and (3) further explain the physical meaning of $\Delta\eta$ as a global, wavefield-integrated geometric observable rather than a proxy for broadband coherence loss. Additional analyses and tests have been incorporated to address the concerns from source variability, distinction from waveform coherence, and spatial interpretability. We believe these revisions clarify the value of the method and reinforce its suitability for publication in *Nature Communications*.

Major Point 1: Attribution of $\Delta\eta$ Changes: Subsurface vs. Noise-Source Effects. *While the authors assert that $\Delta\eta$ "remains detectable despite unstable noise fields," the evidence presented does not convincingly distinguish subsurface medium changes from variations in the spatial or temporal distribution of ambient noise sources.*

• Temporal cross-correlation functions (Fig. 4a,d) show clear coherence loss during eruptive phases, likely due to the Mw 6.9 earthquake and elevated seismicity, suggesting that at least part of the $\Delta\eta$ signal could reflect noise-field instability.

• Noise-field localization (Fig. 4c,f) shows significant migration of energy sources before and after eruptions (from summit to distal sites and back). These shifts coincide with the largest $\Delta\eta$ variations, yet no quantitative test demonstrates that $\Delta\eta$ is unaffected by such source evolution.

• Matched Field Processing maps confirm evolving tremor sources, hence please show that comparable $\Delta\eta$ fluctuations would not arise from source movement alone.

• The numerical simulations assume a fixed source distribution, thus testing only sensitivity to medium changes—not to realistic source variability.

Recommendation: Provide a quantitative sensitivity analysis where source geometry and intensity is perturbed under constant medium properties to assess $\Delta\eta$ variability due solely to source changes.

Response: We thank the reviewer for this important comment and apologize for any ambiguity in the original manuscript. We fully agree that changes in $\Delta\eta$ may arise from both subsurface structural changes and the ambient noise source variability. We therefore address this concern quantitatively by separating the analysis into pre-eruptive and co-eruptive regimes.

The Co-eruptive Noise Source Variability vs $\Delta\eta$ Detection

We acknowledge that the evolution of $\Delta\eta$ is influenced by variations in the ambient noise field, particularly during co-eruptive periods characterized by intense seismicity, strong tremor, and large earthquakes. Our intention was not to suggest that $\Delta\eta$ is immune to noise source instability, but rather that $\Delta\eta$ **retains robust and physically interpretable co-eruptive signals even under strongly evolving noise source conditions.** Importantly, during co-eruptive phases, source variability and subsurface medium changes are inherently coupled: eruptive processes simultaneously change waves excitation (e.g., tremor activation, conduit resonance) and structural properties (e.g., fracturing and stress redistribution). In such settings, strict observational separation of source and medium effects is a fundamental challenge for all ambient noise interferometric metrics.

Quantitative Source-Effect Sensitivity Tests

Following the reviewer's recommendation, we performed a dedicated quantitative sensitivity analysis (see an example in Figure R1) in which **source geometry and amplitude are systematically perturbed while the medium properties are fixed.** Such tests are structurally parallel to the $\Delta\eta$ evaluation to subsurface medium changes (Figure 5).

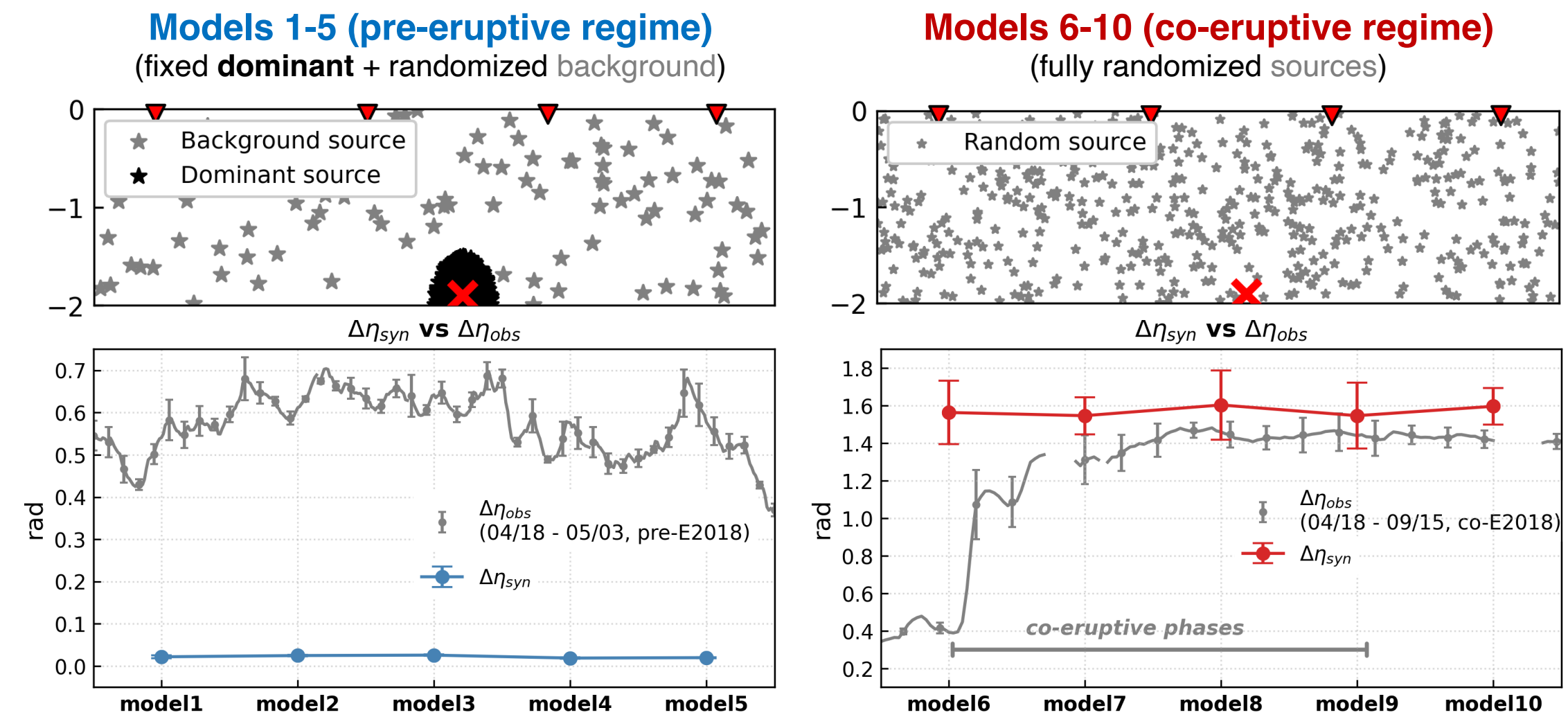

Figure R1: An example of source-effect sensitivity tests in Supplementary Fig. S5. and summarized in Table 1.

Models 1-5 yield $\Delta\eta_{syn}$ values of 0.02-0.03, approximately one order of magnitude smaller than pre-eruptive $\Delta\eta_{obs}$ and $\Delta\eta_{syn}$ based on the medium change (in Figure 5a). Models 6-10 produce $\Delta\eta_{syn}$ values of ~1.5-1.6 comparable to the co-eruptive peak amplitudes, and additionally indicate that $\Delta\eta$ is less sensitive to intensity fluctuation alone but primarily controlled by source geometry reconfiguration. While these tests show that extreme source reconfiguration can generate co-eruptive-level $\Delta\eta$ amplitudes, they are insufficient to explain the structured temporal evolution of $\Delta\eta$ observed during the 2018 eruptive transitions and summit collapse sequence. During co-eruptive periods, elevated seismicity and tremor activity make the ambient noise field difficult to constrain in modeling. The co-eruptive $\Delta\eta$ observations are therefore most consistently interpreted as the coupling effects of source variability and medium structural changes. A strict quantitative decomposition of source and medium effects may require additional explicit time-dependent source imaging and medium inversion.

Performance of $\Delta\eta$ Compared with Conventional Metrics

Despite strong source disturbances during co-eruptive periods, $\Delta\eta$ consistently captures significant 2018 cyclic collapse sequence and the onset and termination of eruptive phases in 2020–2024. In contrast, conventional waveform coherence-based measurements, such as dv/v, are often strongly degraded by source instability and fail in detection, requiring additional stabilization processing.

Related Revision:

- **Table 1 and Supplementary Fig. S5:** We summarize the sensitivity tests of $\Delta\eta$ to source effects into Table 1 in the main text, and provide detailed model configurations and data examples in Supplementary Fig. S5.
- **Line 217:** We add a dedicated subsection "*Assessing geometric phase sensitivity to noise source variability*" to explicitly describe the tests and our interpretations, structurally parallel to the subsequent medium-change sensitivity tests subsection.
- **Line 211-216 and Line 290-292:** We rephrase our previous statement that "$\Delta\eta$ *remains*

detectable despite unstable noise fields" to avoid overstatement. Instead, we specify the performance that $\Delta\eta$ continues to exhibit coherent temporal eruptive signatures under noise field instability.

- **Line 477-481:** We update the model construction for both sensitivity tests in "Methods". Using a consistent reference model from the source-effect tests, we re-run $\Delta\eta$ simulations for magmatic pressure changes, producing similarly robust results in Figure 5 of the main text.

Major Point 2: Demonstrating that $\Delta\eta$ is not a proxy for Coherence Loss.

Figures 4b,e reveal that $\Delta\eta$ extremely closely tracks CCF coherence. The manuscript needs to show what additional information $\Delta\eta$ provides beyond a conventional coherence or waveform-similarity analysis.

• The geometric phase depends on path evolution, similar to waveform similarity, already used in seismic interferometry and non-destructive testing.

• By averaging over station pairs, $\Delta\eta$ sacrifices spatial information that could help disentangle source from medium effects.

• Changes in waveform similarity (and given the correlation also $\Delta\eta$) can reflect either structural perturbations (altered scattering, fracturing) or source variability; this dual sensitivity must be explicitly addressed (see point 1).

Recommendation: Quantitatively compare $\Delta\eta$ to broadband coherence loss and waveform similarity metrics. Clarify whether $\Delta\eta$ captures independent physical information or is a repackaged coherence measure.

Response: We thank the reviewer for highlighting the important concern regarding the distinction of the geometric phase approach. We agree that $\Delta\eta$ correlates with cross-correlation coherence (CC), as it is derived from ambient noise cross-correlations and responds to first-order perturbations of the wavefield. However, $\Delta\eta$ is not defined by waveform similarity, nor is it mathematically equivalent to a coherence measure. CC is a scalar metric that quantifies local waveform similarity. In contrast, **the physical distinction of $\Delta\eta$ lies in its description of the geometric evolution of the global wavefield state, obtained by tracking changes in its trajectory within a Hilbert (parameter) space.** Importantly, our geometric phase sensing framework (Figure 1c) does not explicitly evaluate waveform coherence or similarity. Unlike conventional scalar CC computed independently for station pairs, $\Delta\eta$ is formulated within a vectorial reference-station framework that encodes the global configuration of the wavefield rather than pairwise similarity. $\Delta\eta$ is therefore expected to be more robust to incoherent, local waveform corruption (see the spike-noise tests below). A similar relationship exists between dv/v and CC, which are often correlated but interpreted as complementary rather than redundant measures. We compare $\Delta\eta$ and CC in Figure 4 in this same spirit.

Spike-Noise Tests

Following reviewer's recommendation, we perform controlled spike noise tests (see an example in Figure R2) to evaluate the robustness of both metrics. Artificial spikes with increasing number

and amplitude were randomly inserted into all temporal cross-correlation functions to simulate impulsive, transient disturbances that provide a more stringent test than Gaussian noise. We quantitatively evaluate the responses of CC and $\Delta\eta$ using three statistical metrics: Cohen's D to quantify eruption–background signal separation, RMS to assess absolute deviation from clean results, and NRMS to enable scale-independent comparison. As expected, spike perturbations directly degrade waveform similarity and reduce CC amplitudes. In contrast, $\Delta\eta$ remains comparatively stable and preserves clear eruption–background separation, consistent with its interpretation as a descriptor of global wavefield evolution rather than local waveform similarity.

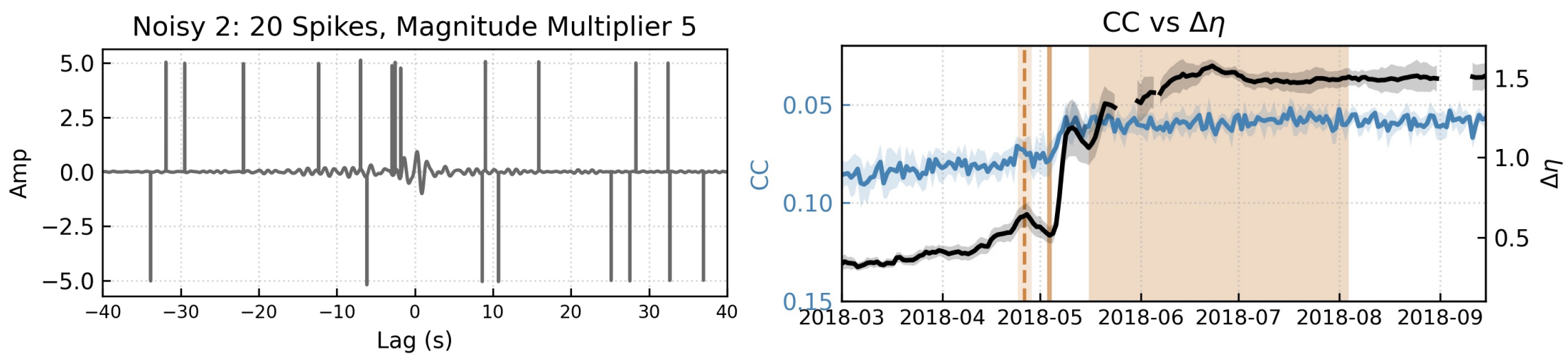

Figure R2: An example of spike-noise tests in Supplementary Fig. S2.

Applications in Non-Destructive Testing

Our project (NewFoS: `https://newfos.arizona.edu/`) aims to broaden the impact of geometric phase across diverse scientific communities, demonstrating that the method is not restricted to volcanic environments but captures structural evolution in diverse wave-supporting systems. Here are two representative publications, as referenced in "Introduction":
—- *Zhang et al., Defect localization in heterogeneous plate structures using the geometric phase change – index of Lamb waves https://doi.org/10.1016/j.ultras.2025.107654.*
—- *Ho et al., Geometric phase sensing using cross-correlations for structural anomaly detection under broadband and stochastic excitations. https://doi.org/10.1115/1.4069302.*

Spatial Interpretability

$\Delta\eta$ requires at least 3 stations (2 connected pairs) to sample a vectorial wavefield. We employ a network-averaged $\Delta\eta$ in the main text to emphasize large-scale, robust wavefield behavior. We now have demonstrated the spatial interpretability of $\Delta\eta$ in the additional subarray tests (see response to "Major Point 3" below)

Sensitivity on Source Effects

We further acknowledge that both CC and $\Delta\eta$ may respond to structural changes and source variability. This dual sensitivity is now explicitly addressed in "Major Point 1". Our source-variability tests show that moderate source variability only produces $\Delta\eta$ amplitudes an order of magnitude smaller than pre-eruptive observations. Although extreme source randomization can produce large amplitudes, such unconstrained perturbations can not explain the structured and temporally coherent evolution observed during eruptive phases.

Related Revision:

- **Line 182-191:** We explicitly clarify the conceptual distinction between $\Delta\eta$ and CCF coher-

ence following the first place where we compare them in Figure 4.

- **Supplementary Fig. S2:** We present full details of the spike-noise tests and quantitative robustness metrics.
- **Line 413-416:** We highlight the distinct reference station (vectorial) setting to differ it from conventional array processing.

Major Point 3: Physical interpretability and mechanistic link.

The conceptual step from "geometric phase rotation in Hilbert space" to "magma-induced medium stiffening" remains insufficiently justified.

• The poroelastic model (Eq. 6) is used post hoc to fit observations, but it does not explain how the geometric phase—constructed from complex CCF vectors—relates physically to volumetric strain or pressure-induced velocity changes.

• The conversion from lava lake height to pressure ($\Delta Pm \approx \rho g \Delta h$) and then to $\Delta\eta$ is empirical, lacking a derivation that links $\Delta V/V$ perturbations to measured phase rotations.

Recommendation: Clarify the model connecting seismic velocity perturbations to geometric phase changes. Consider testing subsets of stations to evaluate how spatial averaging affects sensitivity and interpretability.

Response: We thank the reviewer for highlighting the need to clarify the physical interpretation and mechanism of the geometric phase observable.

Physical Interpretation and Simulation Scope

We agree that $\Delta\eta$ should not be interpreted as a direct measurement of magma pressure, volumetric strain or seismic velocity changes. Instead, $\Delta\eta$ is a global wavefield-level observable that quantifies the cumulative geometric phase evolution of the ambient noise Green's function (GF). We clarify that the poroelastic-based simulations (Figure 5) are not a forward derivation of $\Delta\eta$, but a **physically plausible interpretive framework**. The underlying logic is: Pressure-induced velocity perturbations modify the phase of the GFs; the geometric phase formulation captures the cumulative rotation of the complex state vector constructed from these GFs. This simulation is therefore used to evaluate whether realistic medium perturbations produce phase evolutions consistent with interpretive physical processes, rather than to uniquely predict $\Delta\eta$.

We further acknowledge that the Δh–ΔP–$\Delta\eta$ relation provides a first-order physical proxy, not an analytical derivation. An explicit forward model linking geometric phase observables to elastic parameters remains an open theoretical problem, as geometric phase is a global wavefield property emerging from high-order state evolution rather than a local material parameter. Accordingly, our simulations are designed to test sensitivity and physical consistency, demonstrating that medium perturbations of realistic magnitude produce coherent $\Delta\eta$ evolution consistent with observations.

Subarray-level $\Delta\eta$ Sensitivity Tests

As suggested by the reviewer, we evaluate the spatial interpretability of $\Delta\eta$ by using subsets of

stations (Figure R3). This test focuses on the inflation peak on April 26, 2018. We grouped 7 stations into southern and northern subsets (Panel a) and computed $\Delta\eta$ for all possible 5-station subarrays, yielding 21 subarray-level $\Delta\eta$ curves (for each possible reference station) for each northern/southern group (Panel c).

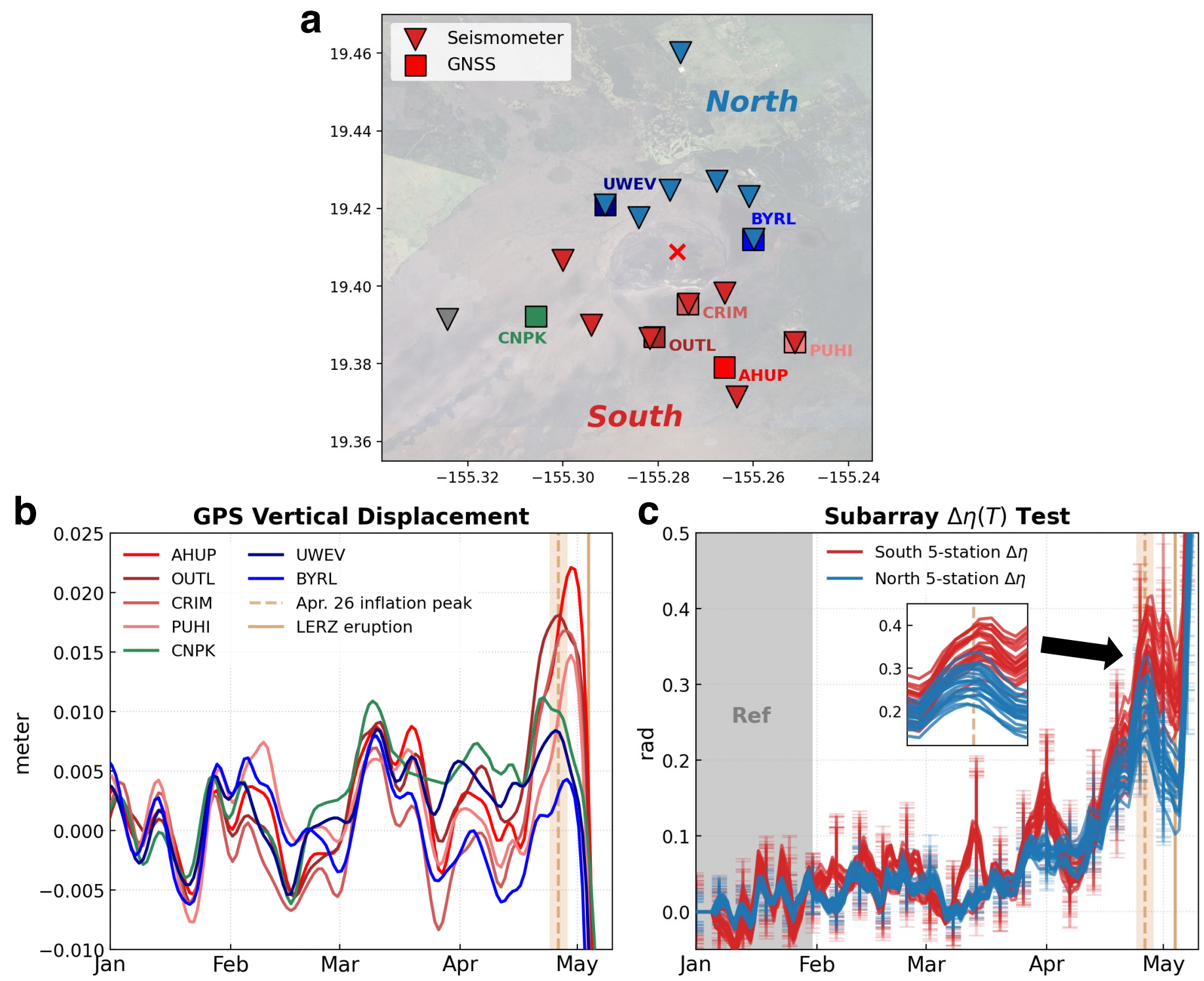

Figure R3: Subarray-level $\Delta\eta$ tests. Same as Supplementary Fig. S11

The southern subarrays (in red) exhibit systematically larger $\Delta\eta$ amplitudes than the northern ones (in blue), consistent with the GPS observations and interpretations of southeastward magma migration toward PuʻuʻŌʻō and LERZ. In addition, the larger variability (higher standard deviation) among the southern subarrays likely reflects more complex inflation and southward intrusive processes. Taken together, this subarray test demonstrates that $\Delta\eta$ can provide spatial interpretability through targeted station subset analyses, rather than solely relying on network-averaged measurements.

More generally, spatial interpretability of $\Delta\eta$ is an active direction of our ongoing work in both seismological sensing and non-destructive testing. Since summer 2024, we have deployed a dense broadband array in a forested area in Fairbanks, Alaska (Network ZG: `https://doi.org/10.7914/x232-db54`), designed to monitor the spatiotemporal permafrost dynamics. This well-configured array will provide an opportunity to further explore the spatial sensing capability of $\Delta\eta$.

Related Revision:

- **Line 250-254:** We clarify the scope of the poroelastic-based simulations.
- **Line 345-354 and Supplementary Fig. S11:** We add an independent paragraph in "Dis-

cussion" to demonstrate the challenge of analytical derivation of $\Delta\eta$ due to its globally integrated nature. We then summarize the subarray tests to indicate the potential avenue for future spatially resolved geometric phase sensing.

Additional Comment 1: *Statistical validation: No quantitative test supports the significance of detected changes. Apply a change-point or time-series analysis (e.g., Sánchez-Pastor 2018) to confirm statistically meaningful shifts.*

Response: Thank you. As our goal is to detect predefined eruptive phases in $\Delta\eta$, we evaluate the statistical separability between eruptive and background periods using Cohen's D metric, which was previously verified in the spike-noise evaluation (Supplementary Fig. S2). All independently documented eruptive intervals (2018 and 2020-2024) were pooled and compared against non-eruptive background periods to quantify the magnitude of eruption-related changes. We calculated across different frequency bands (shown in panel titles in Supplementary Fig. S9b and S10b), and the moderate perturbations of Cohen's D values (~8 in 2018 and ~1 in 2020-2024) indicate the signal variability across these bands. In addition, the relatively low Cohen's D value of 1.05 in the low-frequency 2018 $\Delta\eta$ analysis (Figure R5, in response to "Major 5" by Reviewer 2) further justifies the robustness of the proposed quantification for eruptive signal separation. These analyses provide quantitative support for the significance of the observed $\Delta\eta$ shifts.

Line 335-339: We demonstrate the comparable Cohen's D values, which have been labeled in panel titles in Supplementary Fig. S9b and S10b, across different frequency ranges.

Additional Comment 2: *Comparison to dv/v and waveform similarity methods: The current comparison is limited. The Moving-Window Cross-Spectral (MWCS) technique assumes homogeneous changes and is not ideal here. A frequency–time analysis contrasting reference-biased and unbiased approaches would be more informative (see Obermann and Hillers 2019).*

Response: We thank the reviewer for drawing attention to advanced dv/v methods. We agree that the MWCS-based dv/v approach assumes spatially homogeneous velocity perturbations, which may not strictly hold in complex volcanic environments. In our study, dv/v analysis is included as a reproduced comparison rather than a primary interpretative framework. We adopted MWCS because it is one of the most widely used and conventional dv/v estimations in ambient noise monitoring, providing a standard and broadly comparable baseline. We acknowledge that alternative approaches, including frequency-time and reference-independent formulations, may better accommodate heterogeneous or evolving media.

Line 299-301: We have clarified the limitations of the MWCS-based dv/v analysis and cite the alternative approaches mentioned by the reviewer.

Additional Comment 3: *Figures 2–3: Include noise-power or SNR plots alongside $\Delta\eta$ to show whether phase variations coincide with noise-intensity fluctuations.*

Response: Thank you. We have incorporated example spectrograms as additional panels in **Figures 2 and 3** of the main text. We also label 0.6-1.0 Hz and 1.0-1.2 Hz, denoting the $\Delta\eta$ analyzed frequency bands in both periods. The revised figures now more directly illustrate the temporal relationship between spectral power variations and eruption-related phases, allowing readers to visually assess whether changes in $\Delta\eta$ coincide with fluctuations in noise intensity.

Additional Comment 4: *Short-term analyses: The use of 1-hour CCF stacking requires statistical justification, especially during periods of low coherence.*

Response: Thank you. To evaluate the robustness of short-term $\Delta\eta$ estimates, we compute $\Delta\eta$ time series based on hourly CCFs (currently used), 3-hour and 6-hour moving-stacked CCFs (Figure R4).

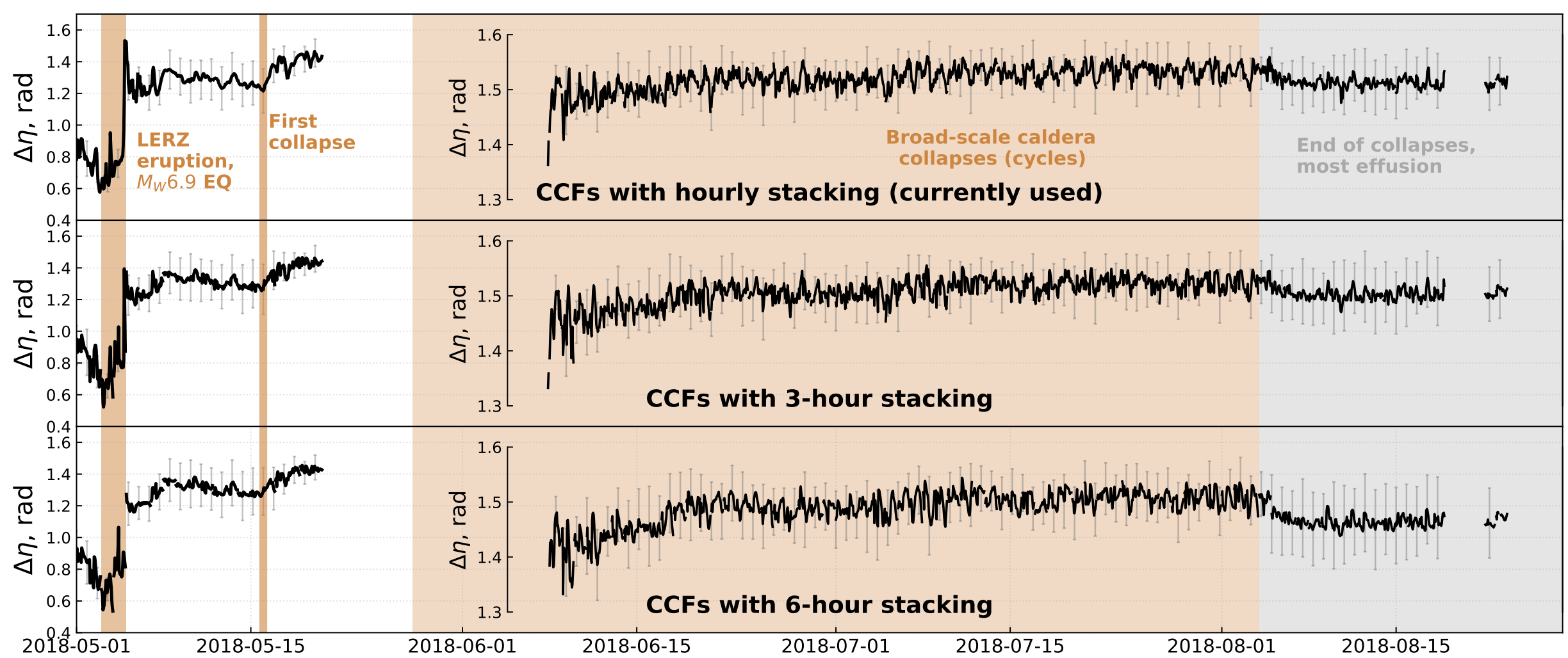

Figure R4: Short-term $\Delta\eta$ analyses based on different moving-stacked CCFs.

All three stacking strategies yield consistent $\Delta\eta$ patterns and successfully capture key co-eruptive signatures, including the two transient signals associated with the LERZ eruption (M_W 6.9 EQ) and the first collapse, as well as the broad-scale collapse sequence. These results indicate that hourly CCF stacking provides a sufficient balance between stable and temporal resolution for short-term $\Delta\eta$ analyses. Longer stacking windows, while further suppressing noise, risk hiding transient changes in the signal. We therefore retain hourly CCFs and consider them justified for resolving short-term eruptive dynamics.

Line 400-402 and Supplementary Fig. S1c: We highlight the CCF stack window test and clarify our selection.

Response to Reviewer 2:

General Comment: *Over the past 10-20 years cross-correlating ambient seismic records for time-lapse monitoring has emerged as a major subdiscipline in seismology. I think a challenge for this field has been that there are so many causes for the types of changes being measured as relative velocity changes, called dv/v. In that way it reminds me of older studies of seismic b-values: so many things can cause variations in it, essentially all subsurface processes one can think of. Studies have shown that relative velocity changes or dv/v can be caused by hydrologic variations, strong ground shaking due to large earthquakes, magma intrusion or extrusion, seasonal or environmental effects, and so on; unraveling which mechanism is the underlying cause for a particular measurement can be difficult.*

In this paper, the authors introduce a new type of processing scheme called geometric phase sensing and apply it to detecting time-lapse changes in ambient seismic records at Kilauea volcano during its recent eruptions. The method is original, interesting, and a potentially paradigm-shifting way of processing ambient noise correlations. However, after reading the paper, I came away with questions and comments that I list in the following. I give the main ones first and then minor ones afterwards.

Response: We thank the reviewer for the thoughtful and constructive overview of the broader context of ambient noise monitoring. We agree that one of the central challenges in time-lapse seismic monitoring lies in disentangling multiple possible physical drivers of observations. In the revised manuscript, we clarify how geometric phase approach relates to and differs from coherence-based metrics, including coherence loss and dv/v measurements. We also expand the discussion of physical interpretation, unique reference setting, and noise-source variability to better position our method within the broader framework of ambient noise monitoring. We appreciate the reviewer's insightful comments, which have helped us improve both the conceptual framing and practical implications of this work.

Major 1: *-My first comment is about the terminology of "geometric phase." This term has already been used in seismology and I'm unclear whether it's being used in the same sense in this paper. For example, the term "geometric phase" is synonymous with "Berry phase" (after the physicist Michael Berry) and has wide applicability in many branches of physics, both quantum and classical. Here is the wikipedia page about it:* `https://en.wikipedia.org/wiki/Geometric_phase`*. In classical physics, a common example of the geometric phase is the motion of a Foucault pendulum due to the Earth's rotation. In seismology, the term "geometric phase" or "Berry phase" has similarly been used to describe changes in seismic waves, in particular the polarization of seismic shear waves, due to the rotation of the Earth or anisotropy. These are a few seismic papers on this phenomenon:*

–Tromp, J. & Dahlen, F. A., 1992. The Berry phase of a slowly varying waveguide, Proc. R. Soc. Lond., A, 437, 329–342.

–Tromp, J. (1994), Surface-Wave Propagation On A Rotating, Anisotropic Earth. Geophysical Journal International, 117: 141-152.

–Snieder, R., C. Sens-Schönfelder, E. Ruigrok, and K. Shiomi (2016), Seismic shear waves as Foucault pendulum, Geophys. Res. Lett., 43, 2576–2581.

When I agreed to review this paper, having only seen the title, I expected something like what I knew as the "geometric phase" in this sense and I'm unsure if the type of analysis in this paper is similar. If it isn't, and my gut feeling is that it is not, then I think there needs to be a statement near the beginning of the paper that clarifies this use of the term "geometric phase" is different from these previous uses. However, given that the term "geometric phase" is already used in seismology, I would recommend changing the terminology if all possible to avoid confusion. An alternative I can think of is that instead of "geometric phase sensing" one could say "topological acoustic sensing." If it is the case that the term "geometric phase" is being used in this paper in a way that this consistent with prior uses of it in seismology, then it's ok to keep using it but again I think there needs to be an explanation early on about how this is connected things like S-wave polarization anomalies due to the Earth's rotation.

Response: We thank the reviewer for this thoughtful comment, and we fully appreciate the potential for confusion arising from the term "geometric phase" (GP). We agree that GP (Berry phase) effects have been previously introduced and studied in seismology, including the works suggested by the reviewer. In these studies, GP arises in association with Earth's rotation or anisotropy and is manifested primarily through shear-wave polarization behavior and related effects.

However, the GP observable ($\Delta\eta$) used in this study is defined and interpreted in a different way. Here, the GP refers to **an accumulated phase extracted from observed seismic wavefields in a non-periodic system**, derived directly from multi-component ambient noise interferometric measurements. **It differs from phase effects along closed or cyclic paths in periodic systems, such as crystals, lattices, and the self-rotated Earth.** Accordingly, the GP used here should not be confused with the Berry phase effects previously discussed in seismology.

In this study, we attempt to develop the term "geometric phase" as an applied solution in an operational and observational sense: it denotes **a directly measurable phase accumulation encoded in the structure of seismic wavefields, which can be continuously tracked in time and leveraged as a practical sensing quantity for environmental monitoring.** While this concept is inspired by

broader developments in geometric and topological wave physics, its formulation and application here are distinct from earlier seismological treatments of geometric (Berry) phase.

Related Revision:

- **Line 71-78**: We have added explicit clarification for our geometric phase ($\Delta\eta$) and cited the previous studies on geometric (Berry) phase in seismology, distinguishing our usage from earlier applications related to Earth's rotation and shear-wave polarization.
- We do not adopt "topological acoustic sensing" as the primary terminology, because it includes a broader class of phenomena. We emphasize "geometric phase" to highlight the specific observable developed and analyzed in this study.

Major 2: *-There is a key reference missing in this paper, which is this one:*

–Muzellec, T., Lesage, P., Caudron, C., & Got, J.-L. (2023). Migration of mechanical perturbations estimated by seismic coda wave interferometry during the 2018 pre-eruptive period at Kīlauea volcano, Hawaii. Journal of Geophysical Research: Solid Earth, 128, e2022JB026224.

I bring it up because in Figure 5 of the paper by Muzellec et al. they show the peak of dv/v occurring on about April 26, 2018 (labeled t1 in their figure) similar to the peak in $\Delta\eta$ (geometric phase change) shown in this paper in Fig. 2. It's important because the authors argue in the Discussion section at lines 249-251 that the observation of this peak in $\Delta\eta$ is an indication of its advantage over dv/v: "For instance, the April 26, 2018 ground inflation peak (Day -4 in Figure 2) was not captured by previous dv/v [42] or our reproduction (2-day lag, inset of Supplementary Fig. S7a), likely due to complex pressure accumulation and reduced bulk edifice strength." In contradiction to this statement, the peak has been captured in a previous dv/v study by Muzellec et al. and they show its exact occurrence in time varies depending on the distance east from the caldera, a precise detail which the authors do not discuss in their paper.

Response: We thank the reviewer for bringing this important reference. We agree that Muzellec et al. (2023) is highly relevant and should be cited and discussed in our manuscript.

In Muzellec et al. (2023), their Fig 5a shows a prominent dv/v peak (labeled $t1$), whose timing depends on station location. As stated in their paper, for stations within or close to the caldera, $t1$ occurs around April 24, while the peak is progressively delayed for stations located further east, with some station pairs exhibiting a peak closer to April 26. We acknowledge that this observation contradicts the overly general statement in our previous "Discussion" that "*the April 26 ground inflation peak was not captured by previous dv/v studies*". We will revise this sentence to clarify that dv/v indeed captures a comparable pre-eruptive signal, but with spatially dependent timing and variability across station pairs.

We emphasize that the advantage of the geometric phase ($\Delta\eta$) observation lies in providing a more temporally coherent indicator that correlates closely with GPS deformation and lava lake dynamics without requiring interpretation of distance-dependent time lags among individual station pairs.

Related Revision:

- **Line 307**: We added this important reference and described the variability of dv/v peak as *"A comparable inflation signal was ..."*.
- **Line 310:** We highlighted the more coherent temporal behavior and higher interpretability of the $\Delta\eta$ evolution as *"In contrast, the temporal evolution of $\Delta\eta$ provides ..."*.

Major 3: *-My overall summary of the geometric phase method is that it is an interesting and fresh approach to time-lapse ambient seismic studies. But there are challenges and tradeoffs.*

•One I can see is that the geometric phase method is very general and one always gets a measurement (a positive advantage), in contrast to dv/v which requires some level of coherence and stability of the noise source. However, there is a sensitivity kernel for dv/v that relates it spatially to physical properties in the subsurface - see the cited paper by Obermann et al. (2013). Obermann et al. also show another independent type of measurement called decorrelation that has a sensitivity kernel related to the changing scattering properties of the subsurface in contrast to changing velocity as for the dv/v. The geometric phase has increased sensitivity compared to dv/v (and presumably decorrelation as well) as the authors show in Fig. 5b, but the tradeoff is that all these different physical changes, like changing velocity or scattering or even changing noise source, are accumulated into $\Delta\eta$. So it will be very difficult, if not impossible, to find meaningful sensitivity kernels for $\Delta\eta$ and to spatially invert for where the changes are happening in the subsurface as can be done for dv/v and decorrelation. So the tradeoff is increased sensitivity to change at the expense of reduced ability to spatially map where the change is occurring.

• It's also interesting to see the similarity between $\Delta\eta$ and the simple correlation coefficient (actually its inverse) as the authors show in their Fig. 4. This shows that $\Delta\eta$ is really a fairly simple measurement similar to the simple correlation coefficient (note that this simple correlation coefficient is different from decorrelation as discussed by Obermann et al.).

•Regarding the need for coherence and noise source stability for dv/v measurements, I'm reminded in a way of similar challenges in InSAR processing in satellite remote sensing. In InSAR, there is a co-registration issue where measurements can't be made if coherence between the two satellite images is lost. This is a similar issue that can afflict dv/v changes and make them impossible to measure. What it makes me wonder is if there may be applications of the geometric sensing method to InSAR processing as well in the future.

So as I said earlier there are tradeoffs that should be acknowledged but the geometric sensing method is a fresh approach and opens up new avenues for further development in the future.

Response: We thank the reviewer for this thoughtful and balanced assessment of the geometric phase approach, which has greatly helped us refine this novel method and enhance its applicability in environmental seismology.

Positive Value of $\Delta\eta$ and Reference State Definition

We agree that $\Delta\eta$ is always mathematically defined with positive values, as it represents the angle evolution of the state vector relative to a chosen baseline. In practice, $\Delta\eta$ is evaluated with respect to a reference state vector constructed from a selected time period. In this study, we define the reference using a one-month relatively quiet pre-eruptive stage to represent a stable background condition. We now explicitly clarified the importance of reference-period selection and its influence on amplitude scaling.

Sensitivity versus Spatial Interpretability of $\Delta\eta$

We agree that $\Delta\eta$ aggregates multiple physical effects along wavepath evolutions into a single global observable. This is an intrinsic consequence of deriving the geometric descriptor of the global wavefield state rather than directly from the perturbation of a specific physical parameter. In this sense, $\Delta\eta$ is conceptually different from the parameter-specific inversion quantity such as dv/v. Like all ambient noise interferometric observables, $\Delta\eta$ relies on seismic cross-correlation (i.e., empirical Green's function approximation) as its foundation. Consequently, it inevitably incorporates untargeted effects, such as noise-source variability, which are intrinsically embedded in the cross-correlation step. We have added noise-source sensitivity tests (referenced below) to quantitatively evaluate the source effect on $\Delta\eta$ in this case study.

We fully acknowledge that this global observable limits the direct derivation of spatially resolved sensitivity kernels comparable to those developed for dv/v or decorrelation (e.g., Obermann et al., 2013). We have clarified this point in the revised manuscript and explicitly state that the current framework is not designed for direct spatial inversion in the same manner as conventional kernel-based approaches. Instead, its strength lies in detecting and characterizing eruptive signals with highly temporal stability and robustness, particularly in regimes where conventional coherence-based methods become unreliable. We further explored the spatial characteristics of $\Delta\eta$ through a supplementary subarray-level test (referenced below). Expanding the spatial interpretability of the geometric phase framework represents an important direction of our ongoing work. As we have deployed a well-configured broadband array in a forested area near Fairbanks, Alaska (Network ZG: `https://doi.org/10.7914/x232-db54`), where we aim to investigate spatiotemporal permafrost dynamics by efficiently including station subset units.

In response to similar concerns raised by Reviewer 1, we have added rigorous tests to clarify the physical distinction, robustness and spatial interpretability of $\Delta\eta$, including:

- **Noise-source tests,** quantifying the sensitivity bounds of $\Delta\eta$ to source variability (response to "Major Point 1" by R1);
- **Spike-noise tests,** examining the robustness and physical distinction of $\Delta\eta$ with respect to waveform coherence (response to "Major Point 2" by R1);
- **Subarray-level $\Delta\eta$ tests,** examining spatial partitioning effects (response to "Major Point 3" by R1).

These tests explicitly demonstrate that while $\Delta\eta$ is sensitive to global wavefield perturbations, its response exhibits physical evolution rather than arbitrary accumulation, and remains stable under incoherent wavefield corruption.

Distinct of $\Delta\eta$ to Waveform Correlation

Although $\Delta\eta$ exhibits empirical correlation with the waveform correlation coefficient (CC) in Figure 4, the two quantities are neither physically redundant nor mathematically equivalent. CC is defined as a scalar measure of local waveform similarity, whereas $\Delta\eta$ is obtained by tracking the evolution of a state vector that encodes the global configuration of the wavefield within a Hilbert (parameter) space. Accordingly, the geometric phase framework does not involve any explicit evaluation of waveform coherence or pairwise similarity. Furthermore, the spike-noise tests demonstrate that $\Delta\eta$ remains stable and preserves eruptive signatures even when CC is substantially degraded by local waveform corruption. We therefore interpret $\Delta\eta$ as a complementary higher-order observable rather than as a proxy for coherence loss. Related concerns have also been addressed in detail in our response to "Major Point 2" by R1.

Potential Application in InSAR:

We thank the reviewer for raising this interesting connection. The "decorrelation challenge" caused by scattering environments or source instability is indeed a fundamental challenge shared by both dv/v and InSAR. While a full exploration is beyond the scope of this seismological study, we offer the following directions on potential cross-disciplinary applications: (1) Wavefield Representation: Our framework tracks the evolution of a state vector that integrates interferometric information. A key question is whether SAR data processing can support a similar vectorial representation of the wavefield to exploit a global geometry. (2) Metric Transformation: Because $\Delta\eta$ provides an independent phase quantification rather than a traditional dynamical phase shift, translating this angular output into surface displacement (the standard InSAR metric) may be a primary challenge. This would likely require an additional inversion processing based on an analytical solution for $\Delta\eta$ within the context of radar backscatter.

Related Revision:

- **Line 322-328:** We have clarified the importance of reference period selection and should be adapted to specific application in "Discussion".
- **Line 345-354:** We have added a paragraph in "Discussion" to clarify that the current tradeoff and challenge of the geometric phase sensing due to its globally integrated nature.
- The noise-source tests, spike-noise tests, and subarray $\Delta\eta$ tests, along with the corresponding revisions, are detailed in our responses to "Major Points 1–3" raised by Reviewer 1.

Major 4: *-The authors point out that there is a discrepancy of 0.3 in their Figure 5 between observed and synthetic $\Delta\eta$, but what are possible explanations for that?*

Response: We thank the reviewer for this question. The ~0.3 discrepancy between observed and synthetic $\Delta\eta$ in Figure 5 is expected given the simplified nature of the simulation model construction. In particular, the ambient noise field is approximated using randomly distributed point sources, the pressure–velocity coupling is implemented using a linear poroelastic strain solution, and the background structure represented by an initial 1-D velocity model could all contribute to the offset. In addition, the magmatic pressure approximation $\Delta P_m(t) \approx \rho_m \cdot g \cdot \Delta h_m(t)$ introduces further

uncertainty. Together, these assumptions define a different baseline for the synthetic geometric phase metric, leading to a systematic offset in $\Delta\eta$ relative to the observations.

Despite these simplifications, we ensure that the synthetic $\Delta\eta$ tests are as consistent as possible with the observations, including the same reference time, the same set of four stations, and the same frequency band (0.6–1.0 Hz). Under these consistent settings, the synthetic results successfully reproduce the first-order temporal evolution of $\Delta\eta$, supporting the interpretation and robustness of the geometric phase sensing approach.

Related Revision:

- **Line 266-270:** We have summarized the possible interpretations for the ~0.3 discrepancy.

Major 5: *-The authors consider several different frequency bands in different parts of the paper like 0.6-1 Hz, 0.5-1.2 Hz, and 1-1.2 Hz. In the supplementary info they show that the precise selection of these bands is not overly critical, which is important since it would be difficult to know the optimal band ahead of time. Have the authors also looked in lower frequency bands that are dominated by classic oceanic microseism noise, like 0.25 Hz and below?*

Response: We thank the reviewer for this question. We agree that the robustness of $\Delta\eta$ with respect to frequency selection is an important aspect of the proposed approach.

Following the reviewer's suggestion, we additionally examine a lower-frequency band of 0.1-0.3 Hz that encompasses classical oceanic microseism energy (Figure R5).

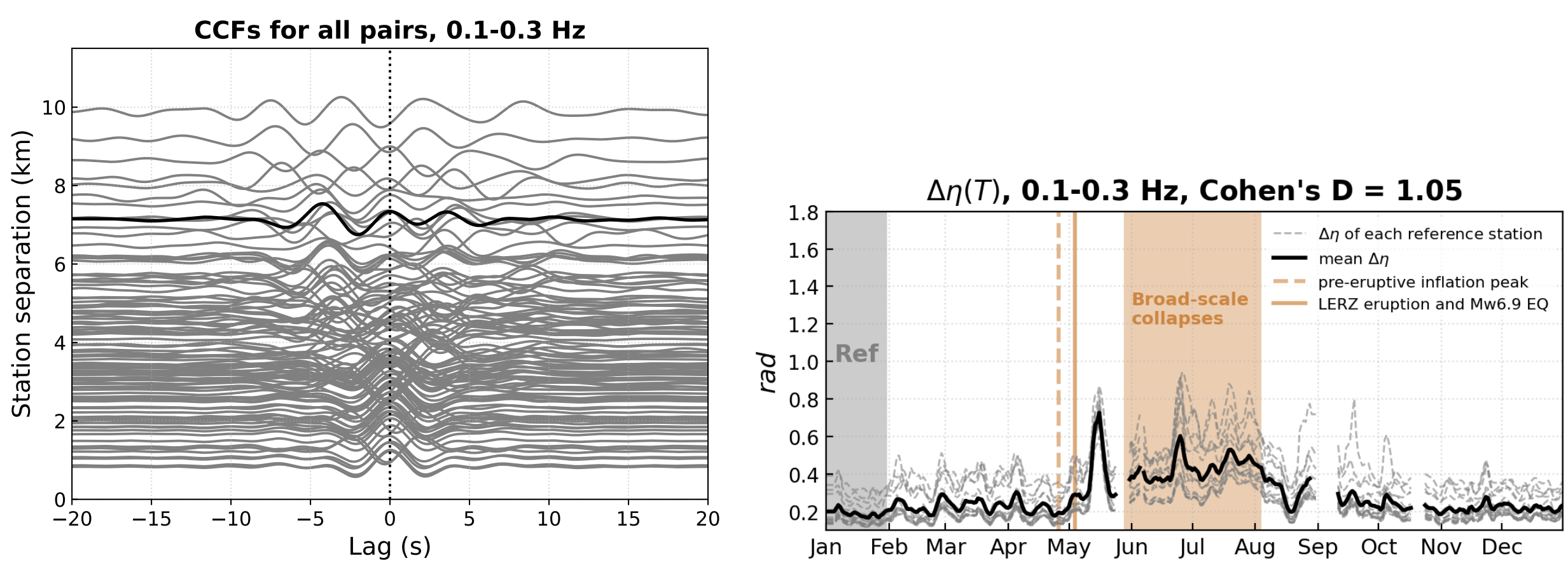

Figure R5: Daily example of all CCFs (left) and resulting $\Delta\eta$ (right) in 0.1-0.3 Hz band.

In this band, the resulting $\Delta\eta$ has smaller values and exhibits a weaker response to eruptive activities compared to the results we displayed from 0.4 to 1.6 Hz. The Cohen's D value, which quantifies the separation between eruption and background signals, decreased from ~8 to 1.05 (compared to selected bands in Supplementary Fig. S9b). This behavior is expected, as lower-frequency seismic waves have much longer wavelengths and sample a larger volume of the medium, reducing

their sensitivity to shallow and localized structural changes associated with magma processes. In the 0.1–0.3 Hz band, Rayleigh-wave wavelengths are approximately 10–30 km (estimated using a representative phase velocity of 3.0 km/s), which greatly exceed the typical inter-station spacing of the array (≤10 km). As a result, phase differences between nearby stations are small, possibly limiting the resolution of $\Delta\eta$ at these frequencies.

Regarding the selection of an optimal frequency range, an advantage of the geometric phase approach is that it naturally produces a time-frequency representation (the direct output of Step 3 in Figure 1c). This allows frequency bands with the most significant and stable $\Delta\eta$ variations to be identified directly throughout a single computation, without repeating tests of individual frequency bands.

Related Revision:

- **Line 336-339:** We describe the weaker responses of $\Delta\eta$ in lower frequencies associated with microseisms as "*Lower-frequency bands dominated by ...*".
- **Line 342-344:** We highlight the optimal frequency determination in the geometric phase framework as "*Importantly, the geometric phase framework ...*"

Minor 1: *Line 158: the term Koa's should instead be Koa'e*

Response: Thank you. In Line165, we have rephrased the word as "Koa'e".

Minor 2: *Line 345: change the word axe to axes*

Response: Thank you. In Line 417, we have rephrased the word as "axes".

Minor 3: *Fig 3 caption: The term "geometric phase" is misspelled. What is meant by the term "future eruptions"? I was confused about what that meant.*

Response: Thank you. We have rephrased the word "geometric phase" in the caption of Figure 3. The two volcano symbols mark the eruption vents of the E2021–2023 event in HC and the E2024 in SWRZ, respectively, which are likely associated with the two intrusive events shown in Panels (b) and (c). We have clarified the timing and location of these eruptions relative to the intrusions in the figure caption.

We thank the reviewers for their careful and constructive evaluation of our manuscript NCOMMS-25-73884A, entitled *"Geometric phase sensing using seismic waves for comprehensive volcano monitoring at Kīlauea Hawaii"*. These insightful comments have significantly helped us improve the clarity, robustness, and presentation of the work. We have revised the manuscript accordingly and provide a detailed, point-by-point response below. Reviewer comments are reproduced in blue italics, and our responses are provided in black.

Response to Reviewer 1:

General Comment: *I thank the authors for their thorough and constructive revision of the manuscript. They have made substantial efforts to address the major concerns raised in the initial review, and the manuscript has improved significantly in rigor and clarity. Importantly, the limitations of the approach are now presented in a transparent manner.*
In my view, the revised version meets the standard for publication. Some interpretational limitations remain, and a fundamental proof of the approach is still lacking; however, these points are now appropriately acknowledged by the authors.
Below, I provide a justification for this assessment based structured along my previously raised major concerns and then a short list of minor revisions that could still be done.

Response: We sincerely thank Reviewer 1 for the careful reevaluation of our revised manuscript and for the positive assessment. We greatly appreciate the reviewer's recognition of the substantial improvements, clarity, and transparency. We are encouraged that the reviewer now finds the manuscript suitable for publication, and we provide point-by-point responses to the remaining minor comments below.

Minor Point 1: *In the subarray analysis section, it should be clearly state that results are indicative only and not equivalent to spatial inversion or localization.*

Response: Thank you. We agree that the subarray analysis should not be interpreted as a formal spatial inversion or localization framework. **In Line 355,** we have revised these summarized sentences *"These subarray-level results should be interpreted ..."* to clarify that the subarray-level $\Delta\eta$ results are intended as qualitative indications of potential spatial sensitivity and partitioning effects, rather than a quantitative spatial inversion or localization.

Minor Point 2: *In the main text and not only the discussion could be stated that co-eruptive $\Delta\eta$ signals remain non-unique and reflect coupled effects*

Response: Thank you. We agree that the coupled-effect sense of co-eruptive $\Delta\eta$ signals should be emphasized more consistently throughout the manuscript. **In Line 153,** we added the clarification *"We emphasize that these co-eruptive $\Delta\eta$ signals are inherently non-unique ..."* immediately when summarizing the 2018 $\Delta\eta$ observations in "Results", to explicitly indicate that the co-eruptive $\Delta\eta$ likely reflects coupled wavefield and structural evolutions.

Minor Point 3: *One explicit sentence could be added acknowledging that $\Delta\eta$ is empirically correlated with coherence, even if conceptually distinct.*

Response: Thank you. **In Line 183,** we added the sentence *"Although $\Delta\eta$ is empirically correlated with ..."* after the comparision between $\Delta\eta$ and coherence in Figure 4 but before the spike-noise test discussion. This statement explicitly acknowledges the empirical correlation between $\Delta\eta$ and waveform coherence, while clarifying the conceptual and mathematical distinction of $\Delta\eta$.

Minor Point 4: *The word "Instead" should be removed from line 75. Or the sentence rephrased.*

Response: Thank you. We have removed "Instead" from the current Line 75.

Response to Reviewer 2:

General Comment: *I have read the authors' responses to my review comments and have come away satisfied that my questions have been addressed properly. I am happy to recommend the manuscript for publication. My only remaining question, based on scientific curiosity, is whether there could be any other useful measurable quantities besides $\Delta\eta$ as defined in equation (2) of the appendix based on this new methodology.*

Response: We sincerely thank Reviewer 2 for the careful reevaluation of our revised manuscript and for the positive recommendation for publication. We greatly appreciate the reviewer's thoughtful comments throughout the review process, which have helped improve the clarity and scientific positioning of this work. We are also encouraged by the reviewer's interest in the broader potential of the proposed methodology, and we respond to the remaining question below.

In Equation 2, we develop $\Delta\eta$ as the primary quantity because the angle metric naturally captures the vectorial evolution and is closely connected to the underlying geometric/topological properties of the framework. We agree that other quantities may also exist within this methodology, including alternative metrics describing the evolution of vectorial or other wavefield representations. Future exploration represents an interesting direction.
